# Comparison of Intermolecular Interactions of Irreversible and Reversible Inhibitors with Bruton’s Tyrosine Kinase via Molecular Dynamics Simulations

**DOI:** 10.3390/molecules27217451

**Published:** 2022-11-02

**Authors:** Xiangfan Yu, Simei Qiu, Dongshan Sun, Pei Guo, Quhuan Li

**Affiliations:** 1 Institute of Biomechanics, School of Bioscience and Bioengineering, South China University of Technology, Guangzhou 510006, China; 2 Guangdong Provincial Engineering and Technology Research Center of Biopharmaceuticals, South China University of Technology, Guangzhou 510006, China

**Keywords:** Bruton’s tyrosine kinase, small molecule inhibitors, molecular simulation, ibrutinib, ARQ531

## Abstract

Bruton’s tyrosine kinase (BTK) is a key protein from the TEC family and is involved in B-cell lymphoma occurrence and development. Targeting BTK is therefore an effective strategy for B-cell lymphoma treatment. Since previous studies on BTK have been limited to structure-function analyses of static protein structures, the dynamics of conformational change of BTK upon inhibitor binding remain unclear. Here, molecular dynamics simulations were conducted to investigate the molecular mechanisms of association and dissociation of a reversible (ARQ531) and irreversible (ibrutinib) small-molecule inhibitor to/from BTK. The results indicated that the BTK kinase domain was found to be locked in an inactive state through local conformational changes in the DFG motif, and P-, A-, and gatekeeper loops. The binding of the inhibitors drove the outward rotation of the C-helix, resulting in the upfolded state of Trp395 and the formation of the salt bridge of Glu445-Arg544, which maintained the inactive conformation state. Met477 and Glu475 in the hinge region were found to be the key residues for inhibitor binding. These findings can be used to evaluate the inhibitory activity of the pharmacophore and applied to the design of effective BTK inhibitors. In addition, the drug resistance to the irreversible inhibitor Ibrutinib was mainly from the strong interaction of Cys481, which was evidenced by the mutational experiment, and further confirmed by the measurement of rupture force and rupture times from steered molecular dynamics simulation. Our results provide mechanistic insights into resistance against BTK-targeting drugs and the key interaction sites for the development of high-quality BTK inhibitors. The steered dynamics simulation also offers a means to rapidly assess the binding capacity of newly designed inhibitors.

## 1. Introduction

BTK (Bruton’s Tyrosine Kinase) is a member of the Tec family of non-receptor Tyrosine kinases. The association of BTK with various diseases has been previously reported [1]. Defects in BTK were identified in primary immunodeficiency X-linked aglobulinemia (XLA) in humans [2] and X-linked immunodeficiency disorders (XID) in mice [3]. XLA is indeed caused by a mutation in the *BTK* gene encoding the BTK protein. This results in the mutation of Arg28 in the BTK structure and further leads to the loss or reduction of mature B cell levels. BTK structure includes pleckstrin homology (PH), Tec homology (TH), Src homology 3 (SH3), Src homology 2 (SH2), and tyrosine kinase (TK) domains [1]. The TK domain includes the kinase active site, which binds ATP at the catalytic rift and phosphorylates signaling molecules. The BTK thereby activates downstream signaling pathways such as the calcium response pathway [4]. The N- and C-lobes of the TK domain are mainly composed of β-sheets and α-helices, respectively. The binding sites of ATP and magnesium ions are located between these lobes. The activation loop [5] (A-loop: Asp539-Phe559) connects the two lobes and regulates binding with downstream molecules. Phosphorylation of Tyr551 at the C-terminal end of the A-loop increases BTK’s activity by ten folds [6,7]. The N-terminus of the A-loop contains a highly conserved aspartic acid-phenylalanine-glycine motif (DFG) whose aspartic acid can be protonated to regulate drug binding [8]. In the N-lobe, the glycine-rich phosphate-positioning loop (P-loop) helps to locate the γ-phosphate group of ATP. The N-lobe contains a conserved lysine (Lys430) that can form hydrogen bonds with ATP’s α-phosphate group. In addition, the N-lobe also has a catalytic helix (C-helix), which contains a conserved glutamate residue (Glu445) that can form a critical salt bridge with Lys430 making BTK in the active state, or with Arg544 keeping BTK in the inactive state [9]. Even these reported researches showed that internal structural changes can affect the functional activity of BTK. Will the conformation of BTK such as A, P-loop, DFG, and salt bridges change after binding inhibitors? And whether the BTK activation has changed accordingly? What are the binding mechanisms of the kinase region of BTK with different inhibitors? This knowledge is not yet known.

BTK is also a tissue-specific protein that is expressed in hematopoietic cells. BTK is involved in physiological processes in B lymphocytes, such as transduction activation, differentiation, proliferation, homing, maturation, and survival [3,10,11]. BTK is an upstream activator of several signaling molecules including NF-κB, AKT, and MYC. Furthermore, BTK plays a role in the BCR pathway and also acts on Toll-like receptors (TLR), chemokine receptors, and various survival-related cytokine receptors [2]. BTK was also found to be a key factor in tumor necrosis factor production in lipopolysaccharide-induced monocytes [12]. Thus, the development of inhibitors targeting BTK has become an important strategy for the treatment of B-cell-related diseases [13,14,15], and even solid tumors [16].

Reversible and irreversible inhibitors of BTK have been identified previously. BTK inhibitors approved by the US FDA are mostly covalent irreversible inhibitors (Table S1), including ibrutinib (2013), acalabrutinib (2017), and zanubrutinib (2020) [12]. Ibrutinib, a first-generation covalent BTK inhibitor, has been widely used in clinics owing to its high inhibitory activity [17]. However, with the widespread clinical application, drug-resistant mutations have emerged in treated patients with chronic lymphocytic leukemia. By deep sequencing of these patients, Cys481 mutation was detected in most patients [18]. A similar pattern of resistance to other covalent inhibitors, such as acalabrutinib, was detected as well [19]. And the acquired resistance due to the C481S mutation in BTK is a major challenge for irreversible inhibitors. Increasing drug resistance has motivated the development of non-covalent reversible BTK inhibitors. The reversible inhibitor ARQ531 was found to maintain inhibitory activity even in the presence of the C481S mutation (Table S2). ARQ531 may therefore provide an alternative treatment option for patients with B-cell malignancies. ARQ531 development has recently entered advanced clinical stages [20,21,22]. Molecular mechanisms underlying the difference in the efficacies of covalent and non-covalent BTK inhibitors remain unclear. Past studies have only focused on clinical aspects and static structure analysis [23,24]. Here, molecular dynamics simulations were conducted to reveal the structural and functional relationship of BTK concerning its interaction with different inhibitors. Our findings are expected to provide theoretical support for the design of novel BTK inhibitors. 

## 2. Results

### 2.1. Conformational Stability of Simulated Systems

**Figure 1:**
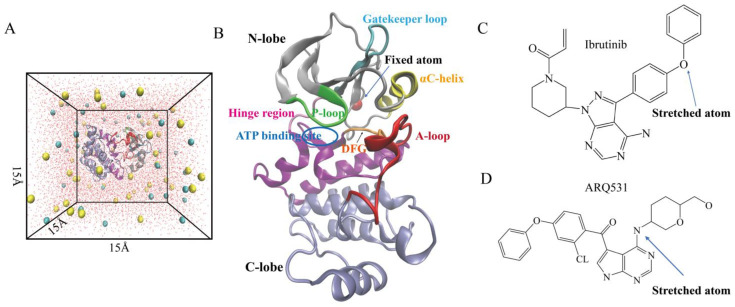
The construction of a simulation system. (**A**) BTK kinase domain with small molecule inhibitors and ions were placed in a 15 × 15 × 15 Å water box. (**B**) Kinase structure. (**C**,**D**) 2D chemical structures of ibrutinib and ARQ531.

**Figure 2:**
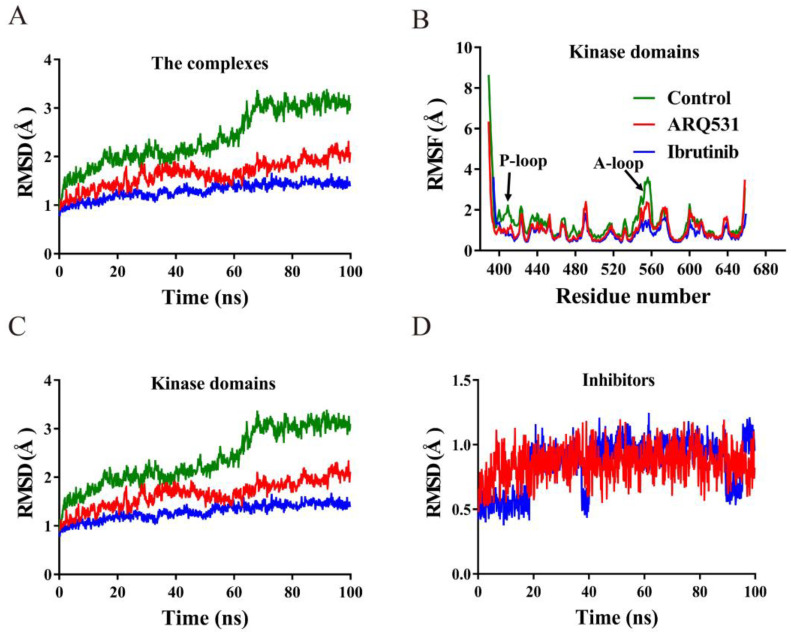
Analysis of conformational stability upon inhibitor binding. (**A**) Time profiles of root mean square deviation (RMSD) values (BTK with Ibrutinib, BTK with ARQ531, BTK without inhibitor). (**B**) Time profiles of per-residue root-mean-square fluctuation (RMSF). (**C**) RMSD time profile of BTK kinase domain. (**D**) RMSD time profiles of small molecule ligands.

We first investigated the conformational stabilities of three simulation systems, including the BTK kinase domain (control), the BTK kinase domain-reversible inhibitor (ARQ531) complex, and the BTK kinase domain-irreversible inhibitor (ibrutinib) complex. The BTK-inhibitor complexes included either ARQ531 or ibrutinib (Figure 1). We calculated the root-mean-square deviation (RMSD) and root-mean-square fluctuation (RMSF) profiles of these systems using simulation trajectories obtained from unrestrained classical MD simulations (Figure 2A,B). The results showed that the binding of small molecule inhibitors enhanced the conformational stability of the BTK kinase domain, as evidenced by stable RMSD time profiles. RMSD profiles of the two inhibitor-BTK complexes were found to be lower than those of the free BTK kinase domain. We also calculated the individual RMSD time profiles of the BTK kinase domain and small molecule inhibitors (Figure 2C,D), and found that conformational changes in the BTK kinase domain were the main contributors to the overall conformation stability. The RMSD values of the BTK kinase domain in the two complexes were below 2 Å (Figure 2C), where the maximum values for systems including reversible (ARQ531) and irreversible (ibrutinib) inhibitors were approximately 1.8 Å and 1.3 Å, respectively. In contrast, the maximum RMSD of the free BTK kinase domain (control) was over 3 Å. In addition, RMSD values of the complex system stabilized below 2 Å in both the reversible inhibitor GDC-0853 and the irreversible inhibitor BGB-3111 (Figure S1). These results show that small-molecule inhibitors increase the conformational stability of the system. We further calculated the per-residue root mean square fluctuation (RMSF) profiles based on Cα atoms of the kinase domain (Figure 2B) and found that inhibitor binding promoted backbone stability, especially around the P-loop (Thr410–Val415) and A-loop (Asp539–Phe559), which showed higher fluctuation levels in the free BTK kinase domain simulation. 

### 2.2. Local Conformational Changes in BTK upon Binding of Different Small Molecule Inhibitors

**Figure 3:**
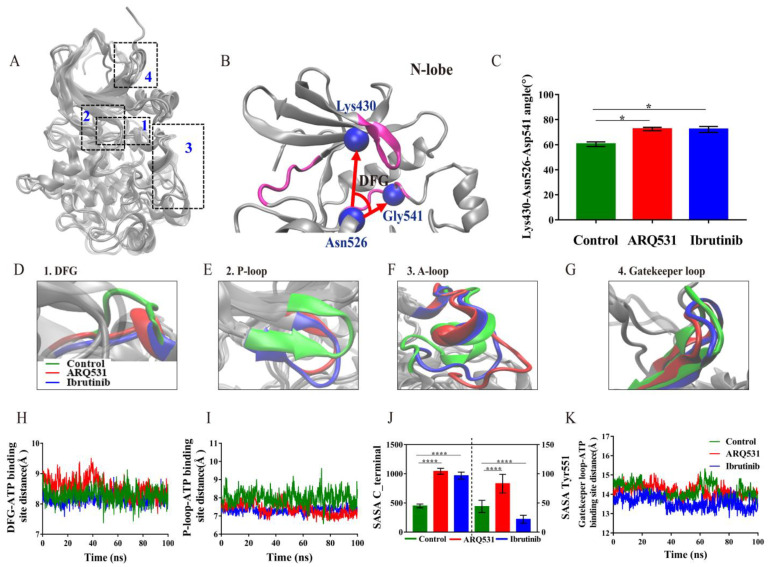
Effects of inhibitor binding on the conformation of ATP binding site. (**A**) Local conformational changes of three simulation systems. 1. DFG motif; 2. P-loop; 3. A-loop; 4. Gatekeeper loop (Residues 462–474). (**B**) Angle between the DFG motif and ATP binding site. (**C**) Changes in angle between the DFG motif and ATP binding site. (**D**–**G**) A close-in on conformations of functional sites on BTK kinase domain. (**H**) Change in distance from DFG to ATP binding site. (**I**) Change in distance between the P-loop and ATP binding site. (**J**) Solvent-accessible surface area (SASA) of the C-terminal helices of A-loop (left) and the phosphorylation site Tyr551 (right). (**K**) Variation in distance between the gatekeeper loop (Residues 462–474) and the ATP binding site. (* *p* < 0.05 and **** *p* < 0.0001).

The results above indicate that the binding of small-molecule inhibitors results in an increase in conformational stability, especially that of the BTK kinase domain. The flexibility analysis also (Figure 2B) showed that the major conformational changes are observed in P-loops (Thr410–Val415) and A-loops (Asp539–Phe559). How do these conformational changes affect the activity of the BTK kinase domain? To answer this question, we superposed the crystal structures of the three systems (Figure 3A) and found four regions with distinct conformations, including the DFG motif, P-loop, A-loop, and the gatekeeper loop. The DFG motif at the N-terminus of the A-loop is a key drug-binding site. We calculated the change in angle (Figure 3B,C) and the distance (Figure 3D,H) between the DFG motif and the ATP binding site upon inhibitor binding. The angle and distance in the free BTK kinase domain were approximately 60° and 8 Å, respectively. In contrast, the angle and distance were slightly higher than 60° and 8 Å in complexes including reversible/irreversible inhibitors. We reason that the conformation of the DFG motif is adjusted to enable stable binding of small-molecule drugs. We also found that the P-loop is oriented inward upon inhibitor binding. Here, the P-loop may act like a door for ATP binding. Indeed, measurements of the distance between the P-loop and ATP binding site revealed that the binding of small molecule inhibitors forced the P-loop to move closer to the ATP binding site, which may result in the prevention of ATP, Ca^2+^, and Mg^2+^ from entering the binding site to activate BTK kinase domain. The P-loop in the complex including the irreversible inhibitor was observed to orient inwards more during the entire simulation, compared to other systems (Figure 3E,I). The A-loop (Figure 3F), was found to be oriented outwards in contrast to the inward orientation of the P-loop. We also measured the solvent-accessible surface area (SASA) of the C-terminal helix and Tyr551 of the A-loop region (Figure 3J). The results showed that the exposure of the C-terminal and Tyr551 phosphorylation sites was increased in the complex including the reversible inhibitor ARQ531, whereas opposite results in Tyr551 phosphorylation sites were observed for the complex including the irreversible inhibitor ibrutinib. Finally, the local conformational analysis revealed that the fluctuation of the gatekeeper loop formed by residues Gly462–Thr474 was higher in inhibitor-BTK kinase domain complexes (Figure 3G,K). Calculations of the distance between the gatekeeper loop and the ATP binding site revealed that the loop was oriented towards the interior of the BTK kinase domain upon inhibitor binding. The role of the gatekeeper region with respect to conformational changes in the BTK kinase domain upon inhibitor binding has not been reported previously in the literature. Hence, we termed it “the gatekeeper” loop. We reason that this gatekeeper loop moves inwards, similar to the closing of a gate, and maybe hinders phosphorylation by the kinase, and thus maintains the inactive conformation of the enzyme. Further research is necessary to determine whether it can be used as a functional target for drug design. In addition, the inactive conformation of BTK protein was also proved to be associated with the DFG motif, P-, A-loop, and the gatekeeper loop in the reversible system GDC-0853 and irreversible system BGB-3111 (Figures S2 and S3). In summary, the BTK kinase domain maintained its inactive conformation upon inhibitor binding by preventing exposure of the ATP binding site and phosphorylation through four key regions: DFG motif, P-loop, A-loop, and the gatekeeper loop.

**Figure 4:**
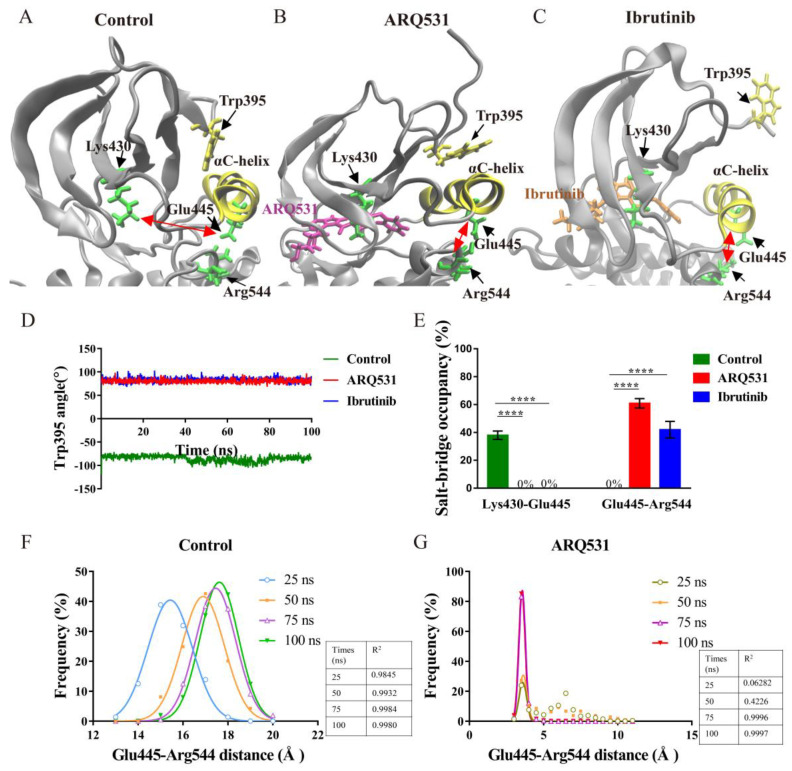
Local conformational changes upon inhibitor binding. (**A**) Conformational changes of the control system (free BTK kinase domain). Double red arrows represent salt bridges. (**B**) Conformational changes in BTK kinase domain upon binding of ARQ531. (**C**) Conformational changes in the BTK kinase domain upon binding of ibrutinib. (**D**) Changes in orientation of Trp395. (**E**) Changes in salt bridges in BTK kinase domain – inhibitor complexes. (**** *p* < 0.0001). (**F**) Changes in the distance between Glu445 and Arg544 in complex including ARQ531. (**G**) Variation in distance between Glu445 and Arg544 in free BTK kinase domain.

The BTK kinase domain was previously found to be activated and inactivated upon the formation of a salt bridge between Glu445 and Lys430, and between Glu445 and Arg544 [25], respectively. We also found that a salt bridge formed between Glu445 and Arg544 in our simulations, which may maintain BTK in an inactive state upon inhibitor binding (Figure 4A–C). How does a salt bridge between Glu445 and Arg544 form, while another salt bridge between Lys430 and Glu445 is broken after inhibitor binding? Conformational analysis showed that the αC-helix was oriented outward upon binding of the inhibitor while driving the rotation of Trp395 and Glu445. A particular finding in this regard was the change in orientation of the Trp395 side chain from a downward-facing to an upward-facing state. Quantification of these changes showed that the angle of Trp395 in the BTK control system was stable at approximately −80°, while the angles of Trp395 in complex systems remained at approximately 80° (Figure 4D). Thus, the BTK kinase domain was maintained in the inactive state, in line with previous experimental results [26]. Another finding was the rotation of the Glu445 side chain, which increased the distance between Glu445 and Lys430, yet decreased the distance between Glu445 and Arg544 to form a new salt bridge in the inhibitor-BTK kinase domain complexes (Figure 4E). Moreover, the salt bridge was maintained for a longer time in the complex including the reversible inhibitor ARQ531 compared to the complex including the irreversible ibrutinib. This may explain the selectivity of reversible BTK inhibition. The distance between Glu445 and Arg544 at four cumulative time points during the simulation was calculated to quantify the conformational change in this regard (Figure 4F,G). The distance between Glu445 and Arg544 was found to decrease gradually over the simulation in BTK kinase domain-inhibitor complexes and finally remained at around 3 Å (Figure 4G). In summary, conformational changes in the αC-helix, Trp395, and the formation of the Glu445-Arg544 salt bridge maintained the BTK kinase domain in an inactive conformation upon inhibitor binding.

### 2.3. Key Intermolecular Interactions in BTK Kinase Domain and Inhibitor Complexes 

**Figure 5:**
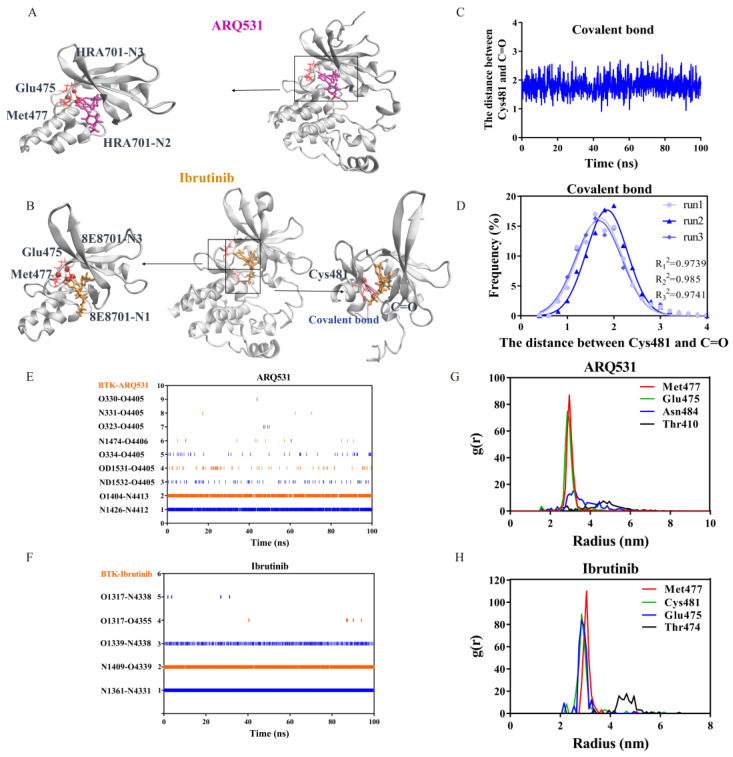
Key interactions between BTK and small molecule inhibitors. (**A**,**B**) Major residues in the interaction interfaces of ARQ531 and ibrutinib with BTK kinase domain as observed in crystal structure. Magenta and orange molecules denote small molecule inhibitors ARQ531 and ibrutinib. Spheres represent atoms, rods represent residues, red lines represent hydrogen bonds, and blue lines represent covalent bonds. (**C**,**D**) Statistics of the mean distance and distance frequency between Cys481 and C=O forming covalent bonds in simulations. (**E**,**F**) Heat map of interacting atomic pairs in complexes including ARQ531 and ibrutinib. (**G**,**H**) Radial distribution function (RDF) of key interaction residues in complexes with ARQ531 and ibrutinib.

The interactions between inhibitors and BTK residues at the binding interface were analyzed. The crystal structures of the complexes formed by BTK and small-molecule inhibitors are shown in Figure 5A,B. The pairs with strong interactions between each other are shown in red, and the covalent bonds are shown in blue. We found that both inhibitors bind to the ATP binding pocket and interact strongly with the hinge region (Figure 5A,B). Strong covalent bond interactions are only observed in the ibrutinib system (Figure 5B). Specifically, covalent bonds were detected between the thiol group of Cys481 of BTK and the α,β-unsaturated ketone of the small-molecule inhibitor. The distance between Cys481 and C=O forming covalent bonds was measured, and the frequency of distance in three independent tests was approximated to a Gaussian distribution. The distance between Cys481 and C=O stabilizes at about 1.9 Å. The R^2^ values of Gaussian fitting for the three statistics were 0.973, 0.984, and 0.973. We use the distance between two atoms to characterize the stability of the covalent bond (Figure 5C,D) because the stable distance between two atoms is conducive to the deprotonation of Cys, the activated Cys amiable nucleus attacks the substrate and promotes the stability of the covalent ligand at the catalytic site.

The residue interaction index, called the RII index, was also calculated based on interactions in the binding interface (Table 1, Figure 5E,F) [27]. Three independent simulations showed that seven interacting residue pairs were formed in the complex including the reversible inhibitor ARQ531, maintaining its bound state. Two residue pairs were found to have relatively persistent high RII values of 0.87 and 0.80 (Table 1, Figure 5E). Only four interacting residue pairs were detected between the BTK kinase domain and the irreversible inhibitor ibrutinib; however, these pairs had RII values of 0.92, 0.81, and 0.72 (Table 1, Figure 5F). We plotted a heatmap of these key interactions in the binding interface as well (Figure 5E,F). The results showed that two pairs of residues were prominent in the interactions between the reversible inhibitor and BTK kinase domain: the N atom of Met477 and N2 atom (index 4412) of the inhibitor, and the O atom of Glu475 and the N3 atom (index 4413) of the inhibitor (Figure 5E). Contacts between three pairs of residues were found to be maintained in the binding interface between the irreversible inhibitor and BTK kinase domain: the N atom of Met477 and the N1 atom (index 4331) of the inhibitor, N atom of Cys481 and O1 atom (index 4339) of the inhibitor, and O atom of Glu475, and N3 atom (index 4338) of the inhibitor (Figure 5F). Using free molecular simulation, combined with mutation techniques, we found that stable covalent and strong hydrogen bond interactions with Cys481 play a very important role in the binding of ibrutinib to BTK. The binding stability and the survival rate of the hydrogen bonds were reduced if destroying the covalent bond by mutating Cys481 (Figure S4D). Our simulation suggested that Cys481 was the most common mutated residue to affect the activity of BTK, and serve as the reason for acquired resistance to ibrutinib [28]. Further calculation of the radial distribution function (RDF) values highlighted the same residues, Met477 and Glu475, in the complex with reversible inhibitor ARQ531 and three residue pairs in the complex with an irreversible inhibitor, including Met477, Cys481, and Glu475 (Figure 5G,H) [29].

### 2.4. Binding Modes and Interaction Energies of Small Molecule Inhibitors

**Figure 6:**
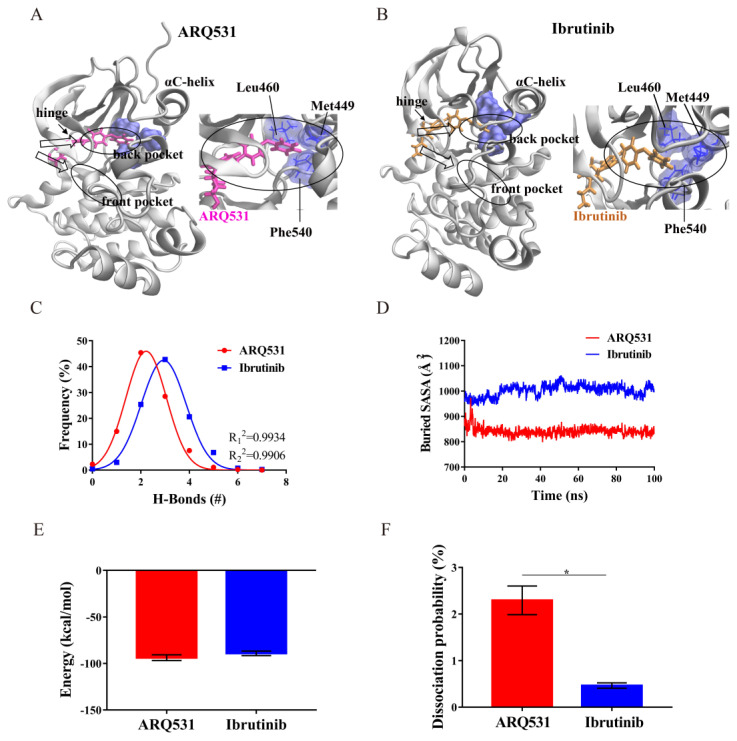
Analysis of binding modes and binding energies of different small molecule inhibitors. (**A**) The binding mode of ARQ531. (**B**) The binding mode of ibrutinib. (**C**) Probability and statistics of hydrogen bond formation in binding interface. (**D**) Solvent accessible surface area (SASA) of the interface. (**E**) Calculation of binding energies in complex systems. (**F**) Probability of hydrogen bond dissociation (* *p* < 0.05).

To further evaluate the mechanism of interaction between BTK and small-molecule inhibitors, we first investigated the binding mode of the two inhibitors and identified that both inhibitors were inserted into the back pocket of the A-loop activation ring and later moved close to the BTK residue Leu460 (Figure 6A,B). This residue is adjacent to Tyr461, a component of the hydrophobic accumulation region. The small molecule inhibitors filling the back pocket may cause conformational adjustment of the hydrophobic accumulation region to trigger the self-inhibited state of BTK, in line with previous literature findings [30]. 

To quantify the intermolecular interactions in whole simulation systems, several parameters were computed, including the H-bond number, buried SASA, interaction energy, and hydrogen bond dissociation probability. First, the distributions of the hydrogen bonds in both inhibitor-BTK kinase domain complexes were determined (Figure 6C). The statistics of the Gaussian fitting curve showed that the number of H-bonds formed by ARQ531 was stable at around 2, and that of ibrutinib was around 3. R^2^ values for both were 0.99. The hydrophobic area of the binding interface was then calculated (Figure 6D). The results revealed a tighter binding interface in the complex with an irreversible inhibitor. The larger the buried SASA value was, the larger the binding surface area was. Next, we calculated interaction energies between small molecules and the BTK kinase domain (Figure 6E). The results showed that the non-covalent interaction energies are essentially the same for both inhibitors. Stable binding results from lower interaction energies in general. Here, a larger RMSD time profile, and more stable interaction was observed for the irreversible inhibitor (Figure 2A). From a theoretical perspective, the interaction energy between an irreversible inhibitor and a BTK kinase domain should be lower than that of a reversible inhibitor. This finding thus raises the question “how do the two systems yield almost identical interaction energies (Figure 6E)?”. This can be explained by the fact that the covalent bonds exist only in the system including the irreversible inhibitor. Even if its contribution to binding does not show up in the interaction energy plot, covalent bonding indirectly affects the probability of hydrogen bond dissociation (Figure 6F). As a result, the probability of hydrogen bond dissociation in the complex with an irreversible inhibitor is smaller than that with a reversible inhibitor. 

### 2.5. Dissociation Kinetics of Inhibitors

The binding affinities of small-molecule inhibitors to BTK depend on binding stability, which arises from the quantity and strength of the interacting residue pairs. Thus, higher binding stability leads to stronger binding affinity. Binding affinity is positively and negatively correlated with association and dissociation rates, respectively. The results shown above are mostly related to factors affecting the association rate, such as the formation of salt bridges (Figure 4) and h-bonds (Figure 5).

**Figure 7:**
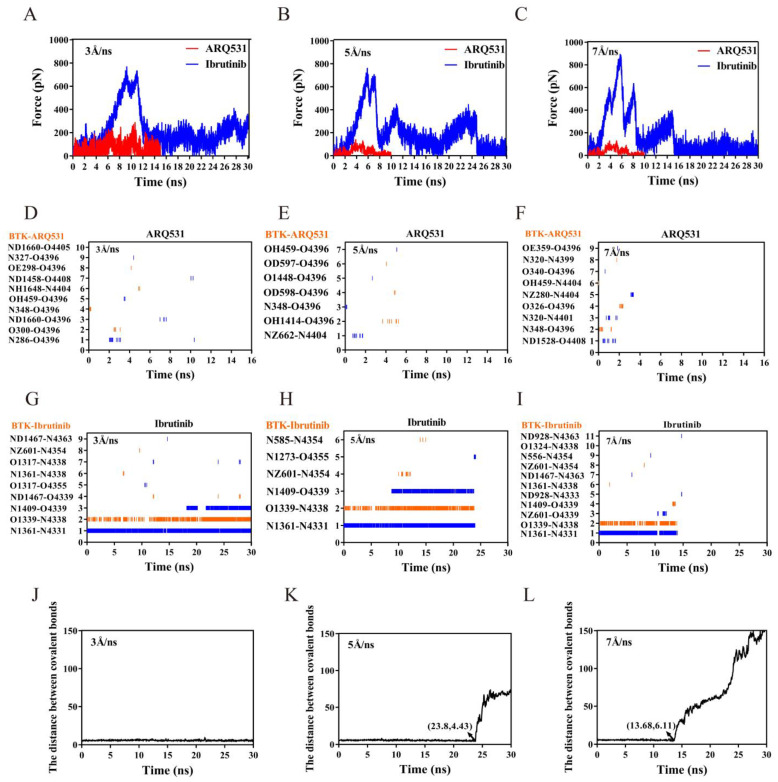
Kinetics of inhibitor dissociation from the complex. (**A**–**C**) Time profiles of force during the dissociation process with constant velocity tensile at 3, 5, and 7 Å/ns velocity. (**D**–**F**) Hydrogen bonds during dissociation of ARQ531 at 3, 5 and 7 Å/ns steering velocity. (**G**–**I**) Hydrogen bonds during dissociation of ibrutinib at 3, 5 and 7 Å/ns steering velocity. (**J**–**L**) The covalent bond dissociation process of ibrutinib at 3, 5 and 7 Å/ns steering velocity.

Other factors related to the dissociation rate should also be investigated to better understand ligand-receptor binding processes. Steered molecular dynamics (SMD) simulations were thus performed to dissociate small-molecule inhibitors from the BTK kinase domain to probe the strength of the inhibitor binding. The more stable conformation in the 100 ns MD simulation trajectory was selected as the initial conformation, and three independent SMDs were performed on the two complex systems with steering velocities of 3 Å/ns, 5 Å/ns, and 7 Å/ns. The force spectrum curve of constant velocity tensile showed a similar trend for the three stretching velocities (Figure 7A–C). The force for the irreversible ibrutinib showed an overall upward trend during the first 5 ns at a tensile speed of 5 Å/ns (Figure 7B). As stretching continued, two consecutive peaks appeared at approximately 6 ns, and the dissociation force reached its maximum level at about 700 pN. During this period, bonds leading to strong resistance were mainly two hydrogen bonds formed between pairs of residues Glu475-N4338 and Met477-N4331. With further stretching, a second peak appeared at 8 ns due to bond fracture, and a third peak was observed at 10 ns. After the last force peak which was observed at 23.8 ns, the inhibitor dissociated completely from the BTK kinase domain (Figure 7B). In contrast, a clear peak at approximately 4 ns was observed, and the rupture force reached a maximum of approximately 100 pN for the reversible inhibitor ARQ531. Either the final dissociation time or the maximum rupture force for the reversible inhibitor ARQ531 was significantly lower than that of the irreversible ibrutinib. Similar findings were found for the dissociation process with 3 Å/ns and 7 Å/ns stretching velocities (Figure 7A,C). 

To analyze the differences in dissociation, we studied the changes in the hydrogen bonds of the complexes including ARQ531 (Figure 7D–F) and ibrutinib (Figure 7G–I). During the dissociation at 5 Å/ns, three pairs of key residues were found to be responsible for the appearance of three peaks, which was consistent with the unrestrained classical MD simulations. The hydrogen and covalent bonds broke simultaneously, indicating dissociation. Therefore, stable hydrogen bonds in the interface between ibrutinib and BTK kinase domain were due to the presence of covalent bonds. Once the covalent bond was broken, the hydrogen bond dissociated as well, (Figure 7H) and the complex disintegrated (Figure 7K). Rapid dissociation of the ARQ531 from the BTK kinase domain was due to the breaking of the hydrogen bonds (Figure 7E). The dissociation characteristics were similar at 3 Å/ns (Figure 7D,G,J) and 7 Å/ns stretching velocities (Figure 7F,I,L). 

**Figure 8:**
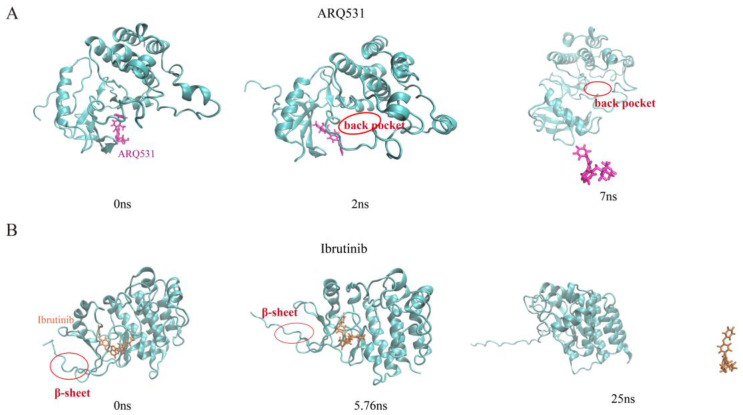
Dissociation pathways of inhibitors (**A**) ARQ531-BTK kinase domain. (**B**) Ibrutinib-BTK kinase domain.

To further understand the dissociation mechanism, conformational changes in the binding interface were analyzed as well (Figure 8). It was found that the complex with the reversible inhibitor was less stable than the complex including the irreversible inhibitor, even though more hydrogen bond interactions were made by the reversible inhibitor. The small-molecule inhibitor, ARQ531, was pulled out from the back pocket of the A-loop activation ring with increasing force. The hydrogen bonds were broken gradually until the complex dissociated completely (Figure 8A). Unlike the reversible inhibitor, the binding of the irreversible inhibitor to the BTK kinase domain was found to be stable due to the existence of covalent and non-covalent bonds. Even when a tensile force was applied, the binding interface between the BTK kinase domain and the small-molecule inhibitor ibrutinib remained intact. The N-terminal β sheets of BTK were unfolded before the hydrogen bonds and covalent bonds that maintained the interaction of the complex were broken (Figure 8B).

### 2.6. Quantification of Dissociation of Different Inhibitors from BTK Kinase Domain

**Figure 9:**
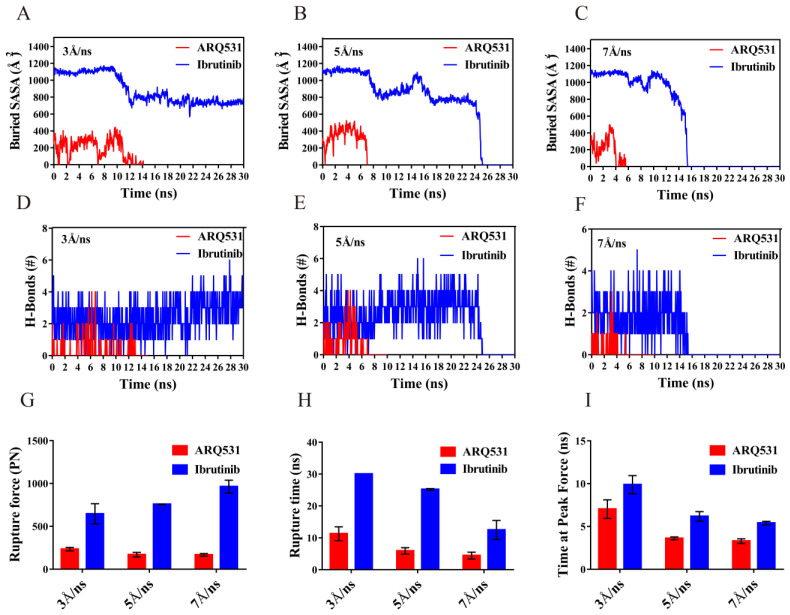
Statistical analysis of dissociation process. (**A**–**C**) The buried SASA value of the interface at 3, 5 and 7 Å/ns steering velocities. (**D**–**F**) The number of hydrogen bonds (H-Bonds) at 3, 5 and 7 Å/ns steering velocities. (**G**) Rupture force. (**H**) Rupture time. (**I**) Breaking time at the maximum peak.

The differences between the dissociation modes of reversible and irreversible inhibitors described above were quantified. For this purpose, we calculated the buried SASA (Figure 9A–C) and hydrogen bonds (Figure 9D–F) for both systems in three independent simulation trajectories. The solvent-inaccessible surface area, also known as the binding surface of the complex, was represented by the buried SASA value. This value was found to be significantly higher for the irreversible inhibitor ibrutinib than the reversible inhibitor ARQ531. At a tensile speed of 5 Å/ns, the buried SASA value of the complex including the irreversible inhibitor showed a brief initial plateau, followed by a downward trend (from approximately 1100 Å^2^ to approximately 800 Å^2^), as the tensile force was increased. Two other plateaus occurred at 10 ns and 18 ns, suggesting that strong interactions between inhibitors and the BTK kinase domain disappeared after plateaus were observed. The values of buried SASA as well as the number of hydrogen bonds dropped to zero at 25 ns (Figure 9B,E), and complete dissociation occurred. For the reversible inhibitor at a tensile speed of 5 Å/ns, the buried SASA value of the complex decreased from 400 Å^2^ to 0 within 7 ns (Figure 9B), and the number of hydrogen bonds decreased gradually to 0 at 7 ns (Figure 9E); and finally the complex dissociated completely (Figure 7E). The results showed that the irreversible inhibitor is more difficult to dissociate than the reversible inhibitor. In contrast, the buried SASA value and hydrogen bond of the binding surface remained stable until the end of the 30 ns-simulation processes at a tensile speed of 3 Å/ns (Figure 7A,D). However, the buried SASA value and hydrogen bond rapidly dropped to zero due to the dissociation of the complex at a tensile speed of 7 Å/ns (Figure 7C,F). These two parameters are closely related to dissociation. We further calculated the rupture force (Figure 9G) and rupture time (Figure 9H) from the time profiles of force during the dissociation process. The results showed that the rupture force increased with the tensile velocity (Figure 9G). However, the rupture time and the time required to reach the maximum peak decreased with an increase in velocity (Figure 9H,I). Both rupture force (Figure 9G) and rupture time (Figure 9H) were smaller for the reversible inhibitor compared to the irreversible inhibitor. In particular, the complex did not dissociate at a relatively low tensile speed (such as 3 Å/ns in this system), which was not sufficient to break the bonds between the irreversible inhibitor and the BTK kinase domain within 30 ns (Figure 7A). However, in the irreversible system with the Cys481 mutation, we found that the breakdown of the covalent bond caused the complex to dissociate at 5ns under a stretching speed of 5 Å/ns, and the rupture force was less than 200 pN (Figure S4).

## 3. Discussion

Small molecule inhibitors of BTK suffer from problems such as drug resistance and off-target effects. The development of small molecule inhibitors of BTK with high inhibitory activity and high selectivity remains a challenge. Here, irreversible [31] and reversible inhibitors [32] were investigated to reveal differences between their BTK binding mechanisms. Ibrutinib and ARQ531 were picked as the representative irreversible and reversible inhibitors from the list as shown in Table S1, and their inhibitory activity of them was summarized in Table S2. As shown in Table S2, the irreversible inhibitor ibrutinib showed strong inhibitory activity with IC_50_ of 0.5 nM and the reversible inhibitor ARQ531 with IC_50_ of 0.85 nM. The complex including the irreversible inhibitor ibrutinib was found to be more stable than the complex including ARQ531 based on the RMSD values (Figure 2A), the reason was coming from the presence of covalent bonds in the irreversible complex simulation system. Ibrutinib may thus show higher inhibitory activity [33,34]. However, with the mutation at the Cys481 site, the inhibitory activity of the irreversible inhibitor ibrutinib was significantly reduced with IC_50_ of 9.03 nM, while the reversible inhibitor ARQ531 increased its inhibitory activity with IC_50_ of 0.39 nM. Therefore, even if irreversible inhibitors have high inhibitory activity, there are problems of off-target and drug resistance [31,32]. 

In this study, three simulation systems were constructed to study patterns of interaction between inhibitors and BTK. First, we found that inhibitors stabilize the conformation of the BTK. The P-loop in all inhibitor-BTK complexes was oriented inwards and moved closer to the ATP binding site. This may assist in the prevention of ATP, Ca^2+,^ and Mg^2+^ from entering the binding site and promoting protein activation. The A-loop in complexes was far away from ATP binding sites, especially in DFG motifs [6,35], which help stabilize the binding of small-molecule drugs to proteins. In addition, we detected another region, the gatekeeper loop, which includes Thr474 [36], and oriented inwards to prevent protein phosphorylation and maintain the inactive conformation of BTK. Compared to other systems, the RMSD time profile of the complex was lower for the irreversible ibrutinib, the P-loop was closer to the ATP-binding site, and the phosphorylation site Tyr551 was less exposed. These findings indicate that the irreversible inhibitor binds to BTK more strongly, and thereby shows higher inhibitory activity. As for the reversible inhibitor ARQ531, the degree of rigidity was lower, and the interaction formed between residue pairs was less stable. This finding reflects the flexibility of the binding of the reversible inhibitor to the BTK. In addition, we also observed these conformational changes in the complexes, with the irreversible inhibitor BGB-3111 and the reversible inhibitor GDC-0853, which further confirmed our results (Figures S2 and S3).

The key residues involved in the binding of these inhibitors to the hinge region of BTK may be targeted for inhibitor design. A stable covalent bond made by Cys481 and three major non-covalent interacting residue pairs were detected between the irreversible inhibitor and BTK structure. Based on the RDF calculations, Met477, Cys481, and Glu475 were identified as key residues for non-covalent bond formation. In addition, strong hydrogen bond interaction at Cys481 further stabilized the binding of the complex. This is the main reason for drug resistance. Met477 and Glu475 were the main residues showing weak interactions with Cys481 in the reversible inhibitor – BTK complex. In addition, the Asn484 of BTK contributed to the binding of reversible inhibitor ARQ531 and could be considered as a target in inhibitor design. Binding analysis showed that the non-covalent binding energies of the irreversible and reversible systems were not significantly different, but the dissociation probabilities were significantly different. Covalent bonds may thus play an important role in stabilizing ligand binding to BTK.

SMD simulations were conducted at three steering speeds, 3, 5, and 7 Å/ns, to further analyze the mechanism of interaction between the BTK kinase domain and small molecule inhibitors. A larger rupture force and smaller rupture time were observed for the irreversible inhibitor: the rupture force was found to reach 800 pN, in contrast to that for the reversible inhibitor, which remained at 200 pN. With an increase in the steering speed, the rupture time gradually decreased, the rupture force to reach the maximum peak increased, and the required time to reach the maximum peak decreased. This shows that force can affect the binding and dissociation of the complex, and binding is more favorable when the external force is stable within a certain range. Steered molecular dynamic simulation results show that the binding affinity of the irreversible inhibitor to the BTK kinase domain is higher than that of the reversible inhibitor. To further demonstrate the role of covalent bonding, the BTK protein mutated at Cys481 was combined with ibrutinib to achieve energy balance. By analyzing the binding system of the unmutated BTK protein with the irreversible inhibitor ibrutinib, we found that the destruction of covalent bonds reduced the binding stability and the survival rate of the hydrogen bonds, which were generated at the key binding sites Met477, Glu475, and Cys481. (Figure S4D). The influence of covalent bonds cannot be ignored in the binding mechanism as well. Smaller steering speeds (e.g., 3 Å/ns), were not sufficient to break the interactions between the irreversible inhibitor and the BTK kinase domain within 30 ns of simulation (Figure 7A). In summary, our findings here revealed the conformational changes in the BTK kinase domain and the key residues involved in interactions with small-molecule inhibitors. These results provide important information for the further development of high-quality BTK inhibitors. In addition, we found that in the absence of covalent bonds, the dissociation time of the whole mutant system became shorter, and the rupture force decreased. This made the protein and inhibitor less stable (Figure S4), further indicating that covalent bonds play an important role in maintaining binding stability and binding affinity. Interestingly, we also found that the binding affinity of the mutated irreversible system was lower than that of the mutated reversible system.

## 4. Materials and Methods

### 4.1. System Setup

Three systems were constructed and simulated (Figure 1): the kinase domain of the wild-type BTK protein (PDB ID:3K54) [37], the complex of the BTK kinase domain binding with the reversible small-molecule inhibitor ARQ531 (PDB ID:6E4F), and the complex of the BTK kinase domain with the irreversible small-molecule inhibitor ibrutinib (PDB ID:5P9J). The three systems were defined as control (BTK), ARQ531, and ibrutinib (Figure 1B). The PSF file of the control system was generated using the psfgen plug-in of Visual Molecular Dynamics (VMD, version 1.9.2) [38]. The PSF files of inhibitor-BTK kinase domain complexes were generated using CHARMM-GUI [39]. Missing hydrogen atoms were added to the simulation systems using the VMD Autopsf plug-in. Water molecules were added to the systems using the Solvate plug-in to achieve a minimum distance of 15 Å from the protein structure to system boundaries in each direction. TIP3 water model was used (Figure 1A). The system was neutralized with 154 mM sodium and chloride ions using the Autoionize plug-in to mimic the physiological environment. The data and figures of the mutant system are presented in the Supplementary Material. Since the C481S mutation in the BTK kinase domain is the main type of resistance to ibrutinib [18]. The C481S mutation was picked to construct the mutation system of a complex of the irreversible inhibitor ibrutinib in this study. The PSF file of the mutant system was synthesized using CHARMM-GUI. The subsequent setup process was the same as that described above.

### 4.2. Molecular Dynamics Simulations

NAMD 2.13 was used for unrestrained and steered molecular dynamics (SMD) simulations [40]. MD simulations were performed using periodic boundary conditions [41], a 2 fs timestep, the particle mesh Ewald (PME) algorithm for electrostatic interactions, and a 12 Å cutoff for electrostatic and van der Waals interactions [42]. The CHARMM36 atomic field and small-molecule field were selected as the system force fields [43]. First, positional restraints were applied on the protein, and water molecules and small-molecule inhibitors were subjected to energy minimization for 15,000 steps. Here, the main objective was to optimize the position of the proteins with respect to those of inhibitors and water molecules. Then, positional restraints were applied on small molecules, and protein and water molecules were subjected to energy minimization for 5000 steps. After that, positional restraints were applied on protein backbone atoms, and the positions of side chains of amino acids were optimized via 15,000 steps of energy minimization. Finally, all atoms were subjected to 15,000 minimization steps to optimize the entire system. The energy-minimized systems were heated gradually first from 0 to 310 K for 0.1 ns, and then equilibrated for 5 ns with pressure and temperature control. The temperature was maintained at 310 K using Langevin dynamics, and the pressure was maintained at 1 atm using the Langevin piston method. The equilibrated structure was used as the initial conformation for subsequent SMD simulations. Unrestrained molecular dynamics simulations were run three times on each equilibrated system for over 100 ns. 

Constant velocity SMD simulation [44,45,46] was adopted for two inhibitor-BTK kinase domain complexes to observe the dissociation of inhibitors from BTK. After 100 ns of equilibration simulation, stable conformations of BTK protein and inhibitors were selected. The complex was then rebuilt by coordinate adjustments, three steps of energy minimization, and 1 ns of equilibration simulation.

The Cα atom of residue Gly389 at the N-terminus of the BTK kinase domain (Figure 1B) was fixed, and atom 4361 of reversible inhibitor ARQ531 (Figure 1C) and atom 4398 of irreversible inhibitor ibrutinib (Figure 1D) were steered away along the direction of the vector formed between positions of these atoms to the fixed Cα atom. Constant tensile speeds of 3 Å/ns, 5 Å/ns, and 7 Å/ns were used. The elastic coefficient of the virtual spring connecting the virtual and controlled atoms (k) was 7 kcalmol^−1^ A^−2^. The simulation of each system was repeated three times, and the data were averaged. 

### 4.3. Data Processing

Molecular visualization and relevant data analysis were completed using VMD and TCL scripts. The root-mean-square deviation (RMSD) of the simulation systems was calculated to characterize the conformational change and stability of the complex structure [47]. The root-mean-square fluctuation (RMSF) of the Cα atoms of the complex was computed to quantify per-residue flexibility levels [48]. Local conformational changes were quantified based on angles and distance between relevant atoms of the systems. The ATP binding site of the BTK kinase domain is composed of three motifs, including the DFG motif chelating Mg^2+^, P-loop binding the ATP phosphate, and the hinge region binding ATP adenosine [5]. A probe radius of 0.14 nm was used to calculate the solvent-accessible surface area (SASA) [49], which is used to evaluate the level of exposure of amino acids to the solvent. Larger SASA values indicate that residues are more exposed to water, and the area of the binding interface is thus smaller. Hydrogen bonds were detected based on the following criteria: donor-acceptor distance less than 0.35 nm and the donor-hydrogen atom-acceptor angle less than 30. A salt bridge is formed when the distance between the nitrogen atom of the basic residue and the oxygen atom of the acid residue is less than 0.4 nm. The hydrogen bond and salt-bridge persistence levels were calculated as the ratio of the number of conformations in which these interactions were observed to the total number of conformations. Binding energy was calculated using NAMD and CHARMM field files [40]. The rupture time and the maximum rupture force of the time profile of were used to evaluate the affinity between the receptor and the ligand. Statistical differences between the two groups were determined using an unpaired two-tailed Student’s *t*-test. Differences were considered statistically significant when the *p* value was <0.05.

## 5. Conclusions

Due to the problems of drug resistance and low inhibitory activity of existing BTK inhibitors, there is an urgent need to develop high-quality BTK inhibitors. Thus, the study of the interaction mechanism between BTK and inhibitors has become very important. In our study, we found that the inhibitory activity of BTK inhibitors was due to the locking of the active site through local conformations, such as the DFG motif, P-, A-, and gatekeeper loops. Others, the residues of Met477 and Glu475 in the hinge region were proved to be the key residues for inhibitor binding. These findings can be used to evaluate the inhibitory activity of the pharmacophore and apply it to the design of effective BTK inhibitors. In addition, covalent interactions, stronger hydrogen bond interactions, and larger binding areas can increase binding affinity. However, the covalent and strong hydrogen bond interactions at Cys481 could lead to drug resistance when mutated at this site. In comparison, reversible inhibitors retained binding affinity with BTK because Cys481 was not an essential interaction site and the hydrogen bond interaction was weak at this site. These findings may provide useful information for designing BTK inhibitors for the drug-resistant Cys481 mutation. Dissociation kinetics showed that reversible inhibitors were more likely to dissociate from the complex due to their binding flexibility, and the irreversible inhibitor ibrutinib had a greater binding affinity. Therefore, dissociation kinetics can be applied to rapidly evaluate the binding capacity of newly designed inhibitors.

## Figures and Tables

**Table 1:** Residue interaction index (RII) of complexes with ARQ531 and Ibrutinib.

Complex of BTK with ARQ531				Complex of BTK with Ibrutinib			
No	BTK	ARQ531	RII	No	BTK	Ibrutinib	RII
1	Met477	HRA701	0.87	1	Met477	8E8701	0.92
2	Glu475	HRA701	0.80	2	Cys481	8E8701	0.81
3	Asn484	HRA701	0.16	3	Glu475	8E8701	0.72
4	Thr410	HRA701	0.05	4	Thr474	8E8701	0.01
5	Cys481	HRA701	0.02				
6	Leu408	HRA701	0.004				
7	Gln412	HRA701	0.001				

## Data Availability

Not applicable.

## Supplementary Materials

The following supporting information can be downloaded at: https://www.mdpi.com/article/10.3390/molecules27217451/s1, Table S1: Comparison of inhibitory activity between reversible and irreversible inhibitors. Table S2: Comparison of inhibitory activity between irreversible (Ibrutinib) and reversible (ARQ531) inhibitors. Figure S1. Analysis of conformational stability of inhibitors-BTK kinase complexes. Figure S2. Local conformational changes of three simulation systems. Figure S3. Effects of inhibitor binding on the conformation of ATP binding site. Figure S4. Kinetics of dissociation of three systems; References [6,21,50,51,52,53,54,55,56,57,58,59,60,61,62,63,64,65,66,67,68,69,70,71,72,73,74,75,76,77,78].

## Sample Availability

The kinase domain of the wild-type BTK protein (PDB ID:3K54), the complex of the BTK kinase domain with ARQ531 (PDB ID:6E4F), and the complex of the BTK kinase domain with ibrutinib (PDB ID:5P9J) can be downloaded from PDB database (https://www.pdbus.org/). Samples of the simulation compounds are available from the authors.

## References

[1] Mohamed A.J., Yu L., Backesjo C.M., Vargas L., Faryal R., Aints A., Christensson B., Berglof A., Vihinen M., Nore B.F. (2009). Bruton’s tyrosine kinase (Btk): Function, regulation, and transformation with special emphasis on the PH domain. Immunol. Rev..

[2] Tsukada S., Saffran D.C., Rawlings D.J., Parolini O., Allen R.C., Klisak I., Sparkes R.S., Kubagawa H., Mohandas T., Quan S. (2012). Deficient expression of a B cell cytoplasmic tyrosine kinase in human X-linked agammaglobulinemia. 1993. J. Immunol..

[3] Rawlings D.J., Saffran D.C., Tsukada S., Largaespada D.A., Grimaldi J.C., Cohen L., Mohr R.N., Bazan J.F., Howard M., Copeland N.G. (1993). Mutation of unique region of Bruton’s tyrosine kinase in immunodeficient XID mice. Science.

[4] Wang Q., Vogan E.M., Nocka L.M., Rosen C.E., Zorn J.A., Harrison S.C., Kuriyan J. (2015). Autoinhibition of Bruton’s tyrosine kinase (Btk) and activation by soluble inositol hexakisphosphate. Elife.

[5] Qiu S.M., Liu Y.F., Li Q.H. (2021). A mechanism for localized dynamics-driven activation in Bruton’s tyrosine kinase. Roy. Soc. Open Sci..

[6] Hopkins B.T., Bame E., Bajrami B., Black C., Bohnert T., Boiselle C., Burdette D., Burns J.C., Delva L., Donaldson D. (2022). Discovery and Preclinical Characterization of BIIB091, a Reversible, Selective BTK Inhibitor for the Treatment of Multiple Sclerosis. J. Med. Chem..

[7] Dinh M., Grunberger D., Ho H., Tsing S.Y., Shaw D., Lee S., Barnett J., Hill R.J., Swinney D.C., Bradshaw J.M. (2007). Activation mechanism and steady state kinetics of Bruton’s tyrosine kinase. J. Biol. Chem..

[8] Kuglstatter A., Wong A., Tsing S., Lee S.W., Lou Y., Villasenor A.G., Bradshaw J.M., Shaw D., Barnett J.W., Browner M.F. (2011). Insights into the conformational flexibility of Bruton’s tyrosine kinase from multiple ligand complex structures. Protein Sci..

[9] Sultan M.M., Denny R.A., Unwalla R., Lovering F., Pande V.S. (2017). Millisecond dynamics of BTK reveal kinome-wide conformational plasticity within the apo kinase domain. Sci. Rep..

[10] Block H., Zarbock A. (2012). The role of the tec kinase Bruton’s tyrosine kinase (BTK) in leukocyte recruitment. Int. Rev. Immunol..

[11] Singh S.P., Dammeijer F., Hendriks R.W. (2018). Role of Bruton’s tyrosine kinase in B cells and malignancies. Mol. Cancer.

[12] Liu X.J., Liu X., Pang X.J., Yuan X.Y., Yu G.X., Li Y.R., Guan Y.F., Zhang Y.B., Song J., Zhang Q.R. (2021). Progress in the development of small molecular inhibitors of the Bruton’s tyrosine kinase (BTK) as a promising cancer therapy. Biorg. Med. Chem..

[13] Roskoski R. (2016). Ibrutinib inhibition of Bruton’s tyrosine kinase (BTK) in the treatment of B cell neoplasms. Pharmacol. Res..

[14] Narita Y., Nagane M., Mishima K., Terui Y., Arakawa Y., Yonezawa H., Asai K., Fukuhara N., Sugiyama K., Shinojima N. (2021). Phase I/II study of tirabrutinib, a second-generation Bruton’s tyrosine kinase inhibitor, in relapsed/refractory primary central nervous system lymphoma. Neuro Oncol..

[15] Whang J.A., Chang B.Y. (2014). Bruton’s tyrosine kinase inhibitors for the treatment of rheumatoid arthritis. Drug Discov. Today.

[16] Molina-Cerrillo J., Alonso-Gordoa T., Gajate P., Grande E. (2017). Bruton’s tyrosine kinase (BTK) as a promising target in solid tumors. Cancer Treat. Rev..

[17] Akinleye A., Chen Y.M., Mukhi N., Song Y.P., Liu D.L. (2013). Ibrutinib and novel BTK inhibitors in clinical development. J. Hematol. Oncol..

[18] Woyach J.A., Ruppert A.S., Guinn D., Lehman A., Blachly J.S., Lozanski A., Heerema N.A., Zhao W., Coleman J., Jones D. (2017). BTK(C481S)-mediated resistance to Ibrutinib in chronic lymphocytic leukemia. J. Clin. Oncol..

[19] Wu J.J., Zhang M.Z., Liu D.L. (2016). Acalabrutinib (ACP-196): A selective second-generation BTK inhibitor. J. Hematol. Oncol..

[20] Reiff S.D., Mantel R., Smith L.L., Greene J.T., Muhowski E.M., Fabian C.A., Goettl V.M., Tran M., Harrington B.K., Rogers K.A. (2018). The BTK inhibitor ARQ 531 targets Ibrutinib-resistant CLL and Richter transformation. Cancer Discov..

[21] Johnson A.R., Kohli P.B., Katewa A., Gogol E., Belmont L.D., Choy R., Penuel E., Burton L., Eigenbrot C., Yu C. (2016). Battling BTK mutants with noncovalent inhibitors that overcome Cys481 and Thr474 mutations. ACS Chem. Biol..

[22] Reiff S.D., Muhowski E.M., Guinn D., Lehman A., Fabian C.A., Cheney C., Mantel R., Smith L., Johnson A.J., Young W.B. (2018). Noncovalent inhibition of C481S Bruton’s tyrosine kinase by GDC-0853: A new treatment strategy for ibrutinib-resistant CLL. Blood.

[23] Shirley M. (2021). Bruton Tyrosine Kinase Inhibitors in B-Cell Malignancies: Their Use and Differential Features. Target. Oncol..

[24] Jebaraj B.M.C., Muller A., Dheenadayalan R.P., Endres S., Roessner P.M., Seyfried F., Walliser C., Wist M., Qi J., Tausch E. (2022). Evaluation of vecabrutinib as a model for non-covalent BTK/ITK inhibition for treatment of chronic lymphocytic leukemia. Blood.

[25] Marcotte D.J., Liu Y.T., Arduini R.M., Hession C.A., Miatkowski K., Wildes C.P., Cullen P.F., Hong V., Hopkins B.T., Mertsching E. (2010). Structures of human Bruton’s tyrosine kinase in active and inactive conformations suggest a mechanism of activation for TEC family kinases. Protein Sci..

[26] Joseph R.E., Wales T.E., Fulton D.B., Engen J.R., Andreotti A.H. (2017). Achieving a graded immune response: BTK adopts a range of active/inactive conformations dictated by multiple interdomain contacts. Structure.

[27] Liu W., Liu G., Zhou H., Fang X., Fang Y., Wu J. (2016). Computer prediction of paratope on antithrombotic antibody 10B12 and epitope on platelet glycoprotein VI via molecular dynamics simulation. Biomed. Eng. Online.

[28] Bodor C., Kotmayer L., Laszlo T., Takacs F., Barna G., Kiss R., Sebestyen E., Nagy T., Hegyi L.L., Mikala G. (2021). Screening and monitoring of the BTK(C481S) mutation in a real-world cohort of patients with relapsed/refractory chronic lymphocytic leukaemia during ibrutinib therapy. Br. J. Haematol..

[29] Lamichhane T.R., Ghimire M.P. (2021). Evaluation of SARS-CoV-2 main protease and inhibitor interactions using dihedral angle distributions and radial distribution function. Heliyon.

[30] Joseph R.E., Amatya N., Fulton D.B., Engen J.R., Wales T.E., Andreotti A. (2020). Differential impact of BTK active site inhibitors on the conformational state of full-length BTK. Elife.

[31] Dubovsky J.A., Beckwith K.A., Natarajan G., Woyach J.A., Jaglowski S., Zhong Y., Hessler J.D., Liu T.M., Chang B.Y., Larkin K.M. (2013). Ibrutinib is an irreversible molecular inhibitor of ITK driving a Th1-selective pressure in T lymphocytes. Blood.

[32] Liang C., Tian D., Ren X., Ding S., Jia M., Xin M., Thareja S. (2018). The development of Bruton’s tyrosine kinase (BTK) inhibitors from 2012 to 2017: A mini-review. Eur. J. Med. Chem..

[33] Hou K., Yu Z., Jia Y., Fang H., Shao S., Huang L., Feng Y. (2020). Efficacy and safety of ibrutinib in diffuse large B-cell lymphoma: A single-arm meta-analysis. Crit. Rev. Oncol. Hematol..

[34] Deeks E.D. (2017). Ibrutinib: A review in chronic lymphocytic leukaemia. Drugs.

[35] Modi S.J., Kulkarni V.M. (2022). Exploration of structural requirements for the inhibition of VEGFR-2 tyrosine kinase: Binding site analysis of type II, ‘DFG-out’ inhibitors. J. Biomol Struct Dyn..

[36] Estupinan H.Y., Wang Q., Berglof A., Schaafsma G.C.P., Shi Y., Zhou L., Mohammad D.K., Yu L., Vihinen M., Zain R. (2021). BTK gatekeeper residue variation combined with cysteine 481 substitution causes super-resistance to irreversible inhibitors acalabrutinib, ibrutinib and zanubrutinib. Leukemia.

[37] Burley S.K., Berman H.M., Kleywegt G.J., Markley J.L., Nakamura H., Velankar S. (2017). Protein data bank (PDB): The single global macromolecular structure archive. Methods Mol. Biol..

[38] Humphrey W., Dalke A., Schulten K. (1996). VMD: Visual molecular dynamics. J. Mol. Graph..

[39] Jo S., Cheng X., Lee J., Kim S., Park S.J., Patel D.S., Beaven A.H., Lee K.I., Rui H., Park S. (2017). CHARMM-GUI 10 years for biomolecular modeling and simulation. J. Comput. Chem..

[40] Phillips J.C., Braun R., Wang W., Gumbart J., Tajkhorshid E., Villa E., Chipot C., Skeel R.D., Kale L., Schulten K. (2005). Scalable molecular dynamics with NAMD. J. Comput. Chem..

[41] Fang X., Fang Y., Liu L., Liu G., Wu J. (2012). Mapping paratope on antithrombotic antibody 6B4 to epitope on platelet glycoprotein Ibalpha via molecular dynamic simulations. PLoS ONE.

[42] Phillips J.C., Hardy D.J., Maia J.D.C., Stone J.E., Ribeiro J.V., Bernardi R.C., Buch R., Fiorin G., Henin J., Jiang W. (2020). Scalable molecular dynamics on CPU and GPU architectures with NAMD. J. Chem. Phys..

[43] Yu Y., Klauda J.B. (2020). Update of the CHARMM36 united atom chain model for hydrocarbons and phospholipids. J. Phys. Chem. B.

[44] Isralewitz B., Gao M., Schulten K. (2001). Steered molecular dynamics and mechanical functions of proteins. Curr. Opin. Struct. Biol..

[45] Florin E.L., Moy V.T., Gaub H.E. (1994). Adhesion forces between individual ligand-receptor pairs. Science.

[46] Chng C.P., Kitao A. (2010). Mechanical unfolding of bacterial flagellar filament protein by molecular dynamics simulation. J. Mol. Graph. Model..

[47] Sargsyan K., Grauffel C., Lim C. (2017). How molecular size impacts RMSD applications in molecular dynamics simulations. J. Chem. Theory Comput..

[48] Margreitter C., Oostenbrink C. (2017). MDplot: Visualise molecular dynamics. R J..

[49] Wang J., Hou T. (2012). Develop and test a solvent accessible surface area-based model in conformational entropy calculations. J. Chem. Inf. Model..

[50] Honigberg L.A., Smith A.M., Sirisawad M., Verner E., Loury D., Chang B., Li S., Pan Z., Thamm D.H., Miller R.A. (2010). The Bruton tyrosine kinase inhibitor PCI-32765 blocks B-cell activation and is efficacious in models of autoimmune disease and B-cell malignancy. Proc. Natl. Acad. Sci. USA.

[51] Herman S.E.M., Montraveta A., Niemann C.U., Mora-Jensen H., Gulrajani M., Krantz F., Mantel R., Smith L.L., McClanahan F., Harrington B.K. (2017). The Bruton Tyrosine Kinase (BTK) Inhibitor Acalabrutinib Demonstrates Potent On-Target Effects and Efficacy in Two Mouse Models of Chronic Lymphocytic Leukemia. Clin. Cancer Res..

[52] Li N., Sun Z.J., Liu Y., Guo M.M., Zhang Y.L., Zhou D.P., Zhang B., Su D., Zhang S., Han J. (2015). BGB-3111 is a novel and highly selective Bruton’s tyrosine kinase (BTK) inhibitor. Cancer Res..

[53] Xu W., Song Y.P., Li Z.J., Yang S.M., Liu L.H., Hu Y., Zhang W., Zhou J.F., Gao S.J., Ding K.Y. (2019). Safety, Tolerability and Efficacy of Orelabrutinib, Once a Day, to Treat Chinese Patients with Relapsed or Refractory Chronic Lymphocytic Leukemia/Small Cell Leukemia. Blood.

[54] Francesco M.R., Wong M., LaStant J., Finkle D., Loewenstein N., Macsata R., Lindstrom M.M., Shu J., Ton T., Zhu J. (2017). PRN2246, a potent and selective blood brain barrier penetrating BTK inhibitor, exhibits efficacy in central nervous system immunity. Mult. Scler. J..

[55] Evans E.K., Tester R., Aslanian S., Karp R., Sheets M., Labenski M.T., Witowski S.R., Lounsbury H., Chaturvedi P., Mazdiyasni H. (2013). Inhibition of Btk with CC-292 provides early pharmacodynamic assessment of activity in mice and humans. J. Pharmacol. Exp. Ther..

[56] Kokabee L., Wang X., Sevinsky C.J., Wang W.L., Cheu L., Chittur S.V., Karimipoor M., Tenniswood M., Conklin D.S. (2015). Bruton’s tyrosine kinase is a potential therapeutic target in prostate cancer. Cancer Biol. Ther..

[57] Kim Y.Y., Park K.T., Jang S.Y., Lee K.H., Byun J.Y., Suh K.H., Lee Y.M., Kim Y.H., Hwang K.W. (2017). HM71224, a selective Bruton’s tyrosine kinase inhibitor, attenuates the development of murine lupus. Arthritis Res. Ther..

[58] Watterson S.H., Liu Q., Beaudoin B.M., Batt D.G., Li L., Pattoli M.A., Skala S., Cheng L., Obermeier M.T., Moore R. (2019). Discovery of Branebrutinib (BMS-986195): A Strategy for Identifying a Highly Potent and Selective Covalent Inhibitor Providing Rapid In Vivo Inactivation of Bruton’s Tyrosine Kinase (BTK). J. Med. Chem..

[59] Mahajan S., Ghosh S., Sudbeck E.A., Zheng Y., Downs S., Hupke M., Uckun F.M. (1999). Rational design and synthesis of a novel anti-leukemic agent targeting Bruton’s tyrosine kinase (BTK), LFM-A13 [alpha-cyano-beta-hydroxy-beta-methyl-N-(2, 5-dibromophenyl)propenamide]. J. Biol. Chem..

[60] Labenski M., Chaturvedi P., Evans E., Mazdiyazni H., Sheets M., Aslanian S., Nacht M., Petter R., Singh J., Westlin W. (2011). In vitro reactivity assessment of covalent drugs targeting Bruton’s tyrosine kinase. Drug Metab. Rev..

[61] Goess C., Harris C.M., Murdock S., McCarthy R.W., Sampson E., Twomey R., Mathieu S., Mario R., Perham M., Goedken E.R. (2019). ABBV-105, a selective and irreversible inhibitor of Bruton’s tyrosine kinase, is efficacious in multiple preclinical models of inflammation. Mod. Rheumatol..

[62] Caldwell R.D., Qiu H., Askew B.C., Bender A.T., Brugger N., Camps M., Dhanabal M., Dutt V., Eichhorn T., Gardberg A.S. (2019). Discovery of Evobrutinib: An Oral, Potent, and Highly Selective, Covalent Bruton’s Tyrosine Kinase (BTK) Inhibitor for the Treatment of Immunological Diseases. J. Med. Chem..

[63] Pulz R., Angst D., Dawson J., Gessier F., Gutmann S., Hersperger R., Hinniger A., Janser P., Koch G., Revesz L. (2019). Design of Potent and Selective Covalent Inhibitors of Bruton’s Tyrosine Kinase Targeting an Inactive Conformation. ACS Med. Chem. Lett..

[64] Ma B., Metrick C.M., Gu C., Hoemberger M., Bajrami B., Bame E., Huang J., Mingueneau M., Murugan P., Santoro J.C. (2022). Optimization of a novel piperazinone series as potent selective peripheral covalent BTK inhibitors. Bioorg. Med. Chem. Lett..

[65] Ma C., Li Q., Zhao M., Fan G., Zhao J., Zhang D., Yang S., Zhang S., Gao D., Mao L. (2021). Discovery of 1-Amino-1H-imidazole-5-carboxamide Derivatives as Highly Selective, Covalent Bruton’s Tyrosine Kinase (BTK) Inhibitors. J. Med. Chem..

[66] Eathiraj S., Savage R., Yu Y., Schwartz B., Woyach J., Johnson A., Reiff S., Abbadessa G. (2016). Targeting Ibrutinib-Resistant BTK-C481S Mutation with ARQ 531, a Reversible Non-Covalent Inhibitor of BTK. Clin. Lymphoma Myeloma Leukemia.

[67] Erickson R.I., Schutt L.K., Tarrant J.M., McDowell M., Liu L., Johnson A.R., Lewin-Koh S.C., Hedehus M., Ross J., Carano R.A. (2017). Bruton’s Tyrosine Kinase Small Molecule Inhibitors Induce a Distinct Pancreatic Toxicity in Rats. J. Pharmacol. Exp. Ther..

[68] Gomez E.B., Isabel L., Rosendahal M.S., Rothenberg S.M., Andrews S.W., Brandhuber B.J. (2019). Loxo-305, a Highly Selective and Non-Covalent Next Generation BTK Inhibitor, Inhibits Diverse BTK C481 Substitution Mutations. Blood.

[69] Burger J.A. (2014). Bruton’s tyrosine kinase (BTK) inhibitors in clinical trials. Curr. Hematol. Malig. Rep..

[70] Smith P.F., Krishnarajah J., Nunn P.A., Hill R.J., Karr D., Tam D., Masjedizadeh M., Funk J.O., Gourlay S.G. (2017). A phase I trial of PRN1008, a novel reversible covalent inhibitor of Bruton’s tyrosine kinase, in healthy volunteers. Br. J. Clin. Pharmacol..

[71] Ma B., Bohnert T., Otipoby K.L., Tien E., Arefayene M., Bai J., Bajrami B., Bame E., Chan T.R., Humora M. (2020). Discovery of BIIB068: A Selective, Potent, Reversible Bruton’s Tyrosine Kinase Inhibitor as an Orally Efficacious Agent for Autoimmune Diseases. J. Med. Chem..

[72] Xu D., Kim Y., Postelnek J., Vu M.D., Hu D.Q., Liao C., Bradshaw M., Hsu J., Zhang J., Pashine A. (2012). RN486, a selective Bruton’s tyrosine kinase inhibitor, abrogates immune hypersensitivity responses and arthritis in rodents. J. Pharmacol. Exp. Ther..

[73] Liu L., Di Paolo J., Barbosa J., Rong H., Reif K., Wong H. (2011). Antiarthritis effect of a novel Bruton’s tyrosine kinase (BTK) inhibitor in rat collagen-induced arthritis and mechanism-based pharmacokinetic/pharmacodynamic modeling: Relationships between inhibition of BTK phosphorylation and efficacy. J. Pharmacol. Exp. Ther..

[74] Pan Z., Scheerens H., Li S.J., Schultz B.E., Sprengeler P.A., Burrill L.C., Mendonca R.V., Sweeney M.D., Scott K.C., Grothaus P.G. (2007). Discovery of selective irreversible inhibitors for Bruton’s tyrosine kinase. ChemMedChem.

[75] Watterson S.H., De Lucca G.V., Shi Q., Langevine C.M., Liu Q., Batt D.G., Beaudoin Bertrand M., Gong H., Dai J., Yip S. (2016). Discovery of 6-Fluoro-5-(R)-(3-(S)-(8-fluoro-1-methyl-2,4-dioxo-1,2-dihydroquinazolin-3(4H)-yl )-2-methylphenyl)-2-(S)-(2-hydroxypropan-2-yl)-2,3,4,9-tetrahydro-1H-carbazole-8- carboxamide (BMS-986142): A Reversible Inhibitor of Bruton’s Tyrosine Kinase (BTK) Conformationally Constrained by Two Locked Atropisomers. J. Med. Chem..

[76] De Lucca G.V., Shi Q., Liu Q., Batt D.G., Beaudoin Bertrand M., Rampulla R., Mathur A., Discenza L., D’Arienzo C., Dai J. (2016). Small Molecule Reversible Inhibitors of Bruton’s Tyrosine Kinase (BTK): Structure-Activity Relationships Leading to the Identification of 7-(2-Hydroxypropan-2-yl)-4-[2-methyl-3-(4-oxo-3,4-dihydroquinazolin-3-yl)phenyl]- 9H-carbazole-1-carboxamide (BMS-935177). J. Med. Chem..

[77] Buhimschi A.D., Armstrong H.A., Toure M., Jaime-Figueroa S., Chen T.L., Lehman A.M., Woyach J.A., Johnson A.J., Byrd J.C., Crews C.M. (2018). Targeting the C481S Ibrutinib-Resistance Mutation in Bruton’s Tyrosine Kinase Using PROTAC-Mediated Degradation. Biochemistry.

[78] Gui F., Jiang J., He Z., Li L., Li Y., Deng Z., Lu Y., Wu X., Chen G., Su J. (2019). A non-covalent inhibitor XMU-MP-3 overrides ibrutinib-resistant Btk(C481S) mutation in B-cell malignancies. Br. J. Pharmacol..

