# Supplementary material for: Comparison of Intermolecular Interactions of Irreversible and Reversible Inhibitors with Bruton’s Tyrosine Kinase via Molecular Dynamics Simulations
Source: Molecules. 2022 Nov 2;27(21):7451. doi: 10.3390/molecules27217451 (PMC9655453; doi:10.3390/molecules27217451)
Supplement: Supplementary file 1 [file molecules-27-07451-s001.zip › Supplementary Figures S1-S4.pdf]

Supplementary Figures

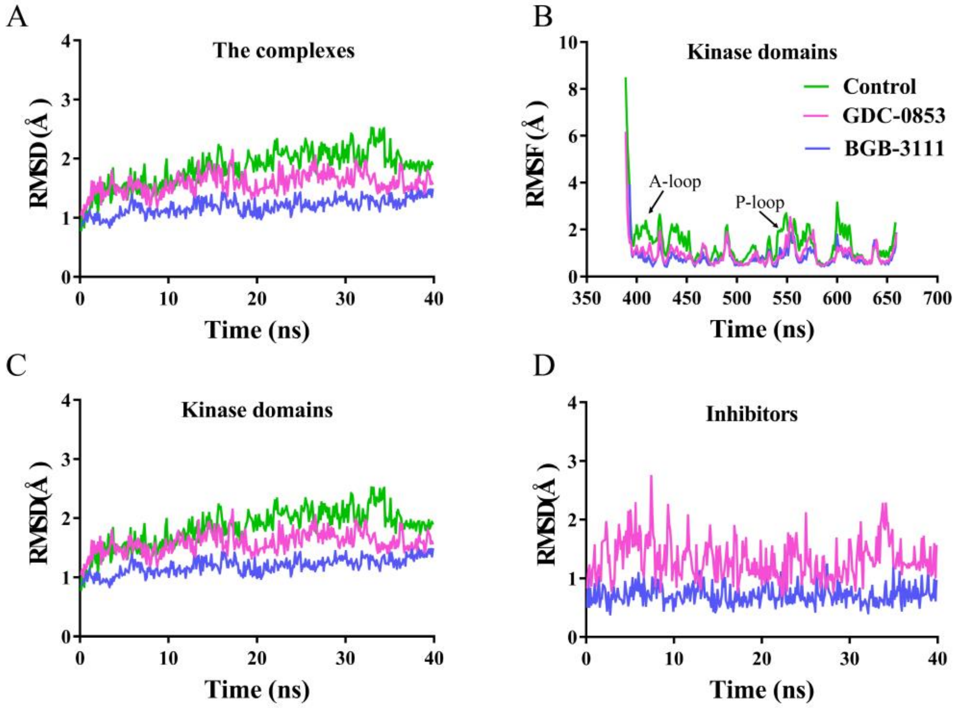

Figure S1. Analysis of conformational stability of inhibitors-BTK kinase complexes. (A) Time profiles of root mean square deviation (RMSD) values of three systems, including WT BTK kinase domains, BTK kinase domains with irreversible inhibitor BGB-3111, and BTK kinase domains with irreversible inhibitor GDC-0853. The short labels of Control, BGB-3111, GDC-0853 were used for these three systems. (B) Time profiles of root-mean-square fluctuation (RMSF). (C) RMSD time profile of BTK kinase domain. (D) RMSD time profiles of small molecule ligands.

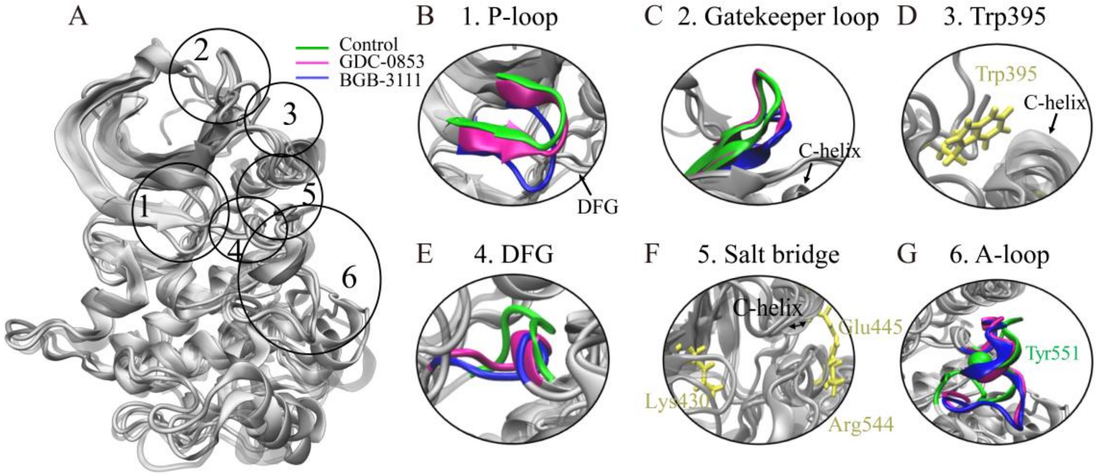

Figure S2. Local conformational changes of three simulation systems. (A)The whole BTK kinase domains of three systems. (B) P-loop. (C) Gatekeeper loop (Residues 462–474). (D) Trp395 (W395). (E) DFG motif. (F) Salt bridge. (G) A-loop.

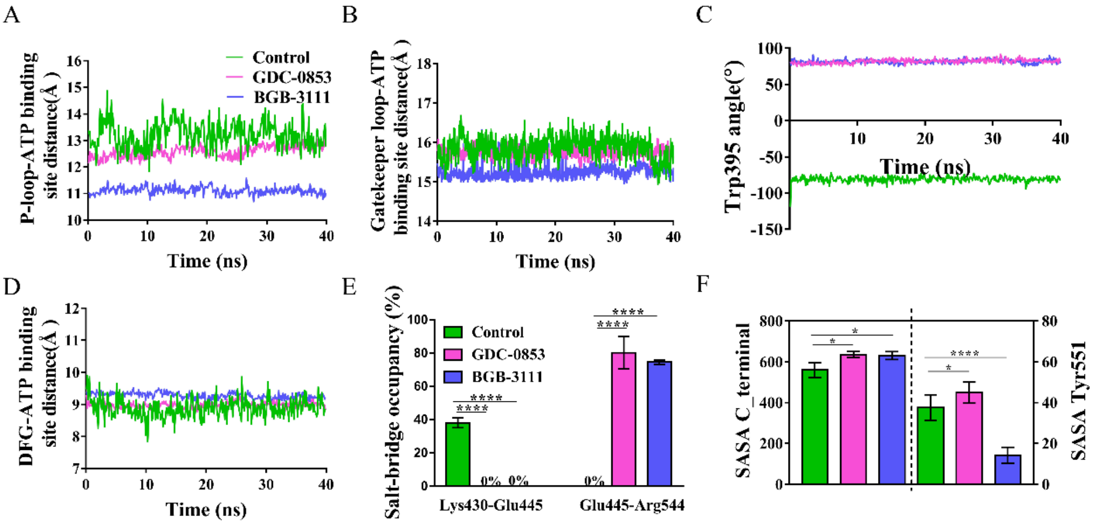

Figure S3. Effects of inhibitor binding on the conformation of ATP binding site. (A) Change in the distance between the P-loop and ATP binding site. (B) Variation in distance between the gatekeeper loop (Residues 462–474) and the ATP binding site. (C) Changes in the orientation of Trp395. (D) Change in distance from DFG to ATP binding site. (E) Changes in salt bridges in BTK kinase domain-inhibitor complexes. (F) Solvent-accessible surface area (SASA) of the C-terminal helices of A-loop (left) and the phosphorylation site Tyr551 (right).

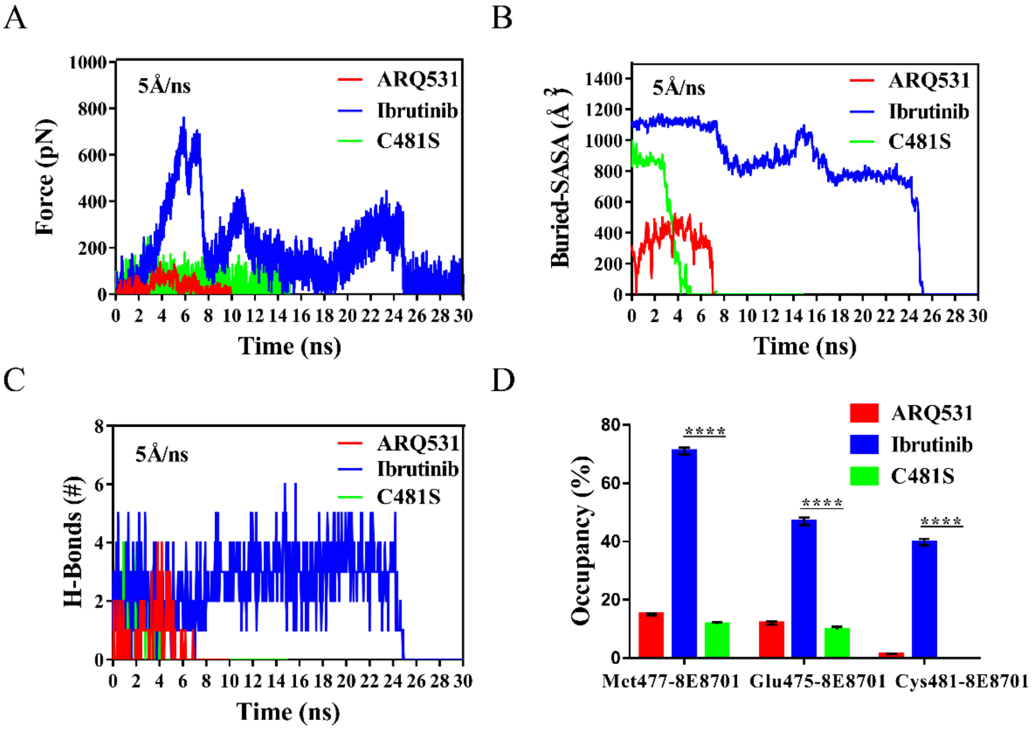

Figure S4. Kinetics of dissociation of three systems. (A) Overall comparison of force spectrum curves of ARQ531, Ibrutinib and C481S (The C481S system came from the irreversible inhibitor Ibrutinib system by mutating Cys481 to Ser481 in BTK kinase domain). (B) Buried SASA value of ARQ531, Ibrutinib, and C481S complex systems. (C) The number of hydrogen bonds present in the interaction surface of ARQ531, Ibrutinib, and C481S complex systems. (D) The survival rates of three pairs of key hydrogen bonds in three complex systems. All data were obtained from tensile dynamics simulation for these three systems under 5 Å/ns steering.
