# Supplementary material for: Comparison of Intermolecular Interactions of Irreversible and Reversible Inhibitors with Bruton’s Tyrosine Kinase via Molecular Dynamics Simulations
Source: Molecules. 2022 Nov 2;27(21):7451. doi: 10.3390/molecules27217451 (PMC9655453; doi:10.3390/molecules27217451)
Supplement: Supplementary file 1 [file molecules-27-07451-s001.zip › Supplementary Table S1.pdf]

Table S1: Comparison of inhibitory activity between reversible and irreversible inhibitors

Inhibitors	Synonymous	IC_{50} (nM)	Clinical stage*	Disease	References
Irreversible inhibitors (Average IC_{50}≈1.28 nM)					
Ibrutinib	PCL-32765	0.5	Stage IV/Stage III	B-cell Lymphoma; Chronic Lymphocytic Leukemia, et al.	[1]
Acalabrutinib	ACP-196	3	Stage III/Stage II	Chronic Lymphocytic Leukemia; Diffuse Large B Cell Lymphoma; COVID-19; et al.	[2]
Zanubrutinib	BGB-3111		Stage III/Stage II	Chronic Lymphocytic Leukemia; B-cell Malignancies; et al.	[3]
Orelabrutinib	ICP-022		Stage II	Mantle Cell Lymphoma; Primary Central Nervous System Lymphoma; et al.	[4]
Tolebrutinib	SAR442168	0.7	Stage II	Primary Progressive Multiple Sclerosis; Relapsing Multiple Sclerosis; et al.	[5]
Spebrutinib	CC-292	0.5	Stage I	Rheumatoid Arthritis; Leukemia Lymphocytic Chronic B-cell; et al.	[6]
CGL-1746		1.9	Candidate drug	B cell-dependent Arthritis	[7]
Poseltinib		1.95	Stage I	Rheumatoid Arthritis; Healthy	[8]
Branebrutinib	BMS-986195	0.1	Stage I	Autoimmune Disorder; Rheumatoid Arthritis; Dermatitis; Atopic	[9]
LFM-A13		2.5	Candidate drug	mouse model of breast cancer	[10]
CNX-774		<1	Candidate drug	Ramos cells; fresh human and rat whole blood	[11]
Elsubrutinib	ABBV-105	0.18	Stage II	Systemic Lupus Erythematosus; Rheumatoid Arthritis	[12]
TL-895		1.5	Clinical	Acute Myeloid Leukemia; COVID-19; Cancer research	[13]
BTK inhibitor 13		1.2	Candidate drug		[14]
BTK-IN-8		0.22	Candidate drug		[15]
BTK inhibitor 19		2.7	Candidate drug		[16]
Reversible inhibitors (Average IC_{50}≈7.17 nM)					
ARQ531		0.85	Clinical	Arthritis	[17]
Fenbrutinib	GDC-0853	0.91(Ki)[&]	Stage III/Stage II	Relapsing Multiple Sclerosis; Urticaria; Systemic Lupus Erythematosuss	[18]
Pirtobrutinib	LOXO-305	3.68	Stage III/Stage II	Chronic Lymphocytic Leukemia; Small Lymphocytic Lymphoma	[19]
BIIB091			Clinical	Autoimmune diseases	[20]
Tirabrutinib	ONO-4059	2.2	Stage II/Stage I	Chronic Spontaneous Urticaria; Sjogren´s Syndrome; Relapsed or Refractory	[21]
Rilzabrutinib	PRN1008	1.3	Stage III/Stage II	Pemphigus; Immune Thrombocytopenia; Warm Autoimmune Hemolytic Anemia	[22]
BIIB068		1	Stage I	Systemic Lupos Erythematosus	[23]
RN486		4	Clinical	Rheumatoid Arthritis; Systemic Lupus Erythematosus	[24]
GDC-0834		5.9	Clinical	Arthritis	[25]
PCL29732		8.2 (Ki)	Clinical	Autoimmune disease; B-cell Malignancy	[26]
BMS-986142		0.5	Stage II/Stage I	Autoimmune diseases	[27]
BMS-935177		3	Clinical	Rheumatoid Arthritis	[28]
N-piperidine ibrutinib hydrochloride		51	Candidate drug		[29]
XMU-MP-3		10.7	Clinical	B-cell Malignancies	[30]

Notes: [&]Ki, inhibition constant. Ki value is equivalent to IC_{50} value to some extent, but it does not change with the change of experimental conditions. *Clinical stage, two clinical stages are the stages in different diseases listed in disease lines

References

1. Honigberg, L. A.; Smith, A. M.; Sirisawad, M.; Verner, E.; Loury, D.; Chang, B.; Li, S.; Pan, Z.; Thamm, D. H.; Miller, R. A.; Buggy, J. J., The Bruton tyrosine kinase inhibitor PCI-32765 blocks B-cell activation and is efficacious in models of autoimmune disease and B-cell malignancy. *Proc. Natl. Acad. Sci. U. S. A.* **2010,** 107, (29), 13075-80.
2. Herman, S. E. M.; Montraveta, A.; Niemann, C. U.; Mora-Jensen, H.; Gulrajani, M.; Krantz, F.; Mantel, R.; Smith, L. L.; McClanahan, F.; Harrington, B. K.; Colomer, D.; Covey, T.; Byrd, J. C.; Izumi, R.; Kaptein, A.; Ulrich, R.; Johnson, A. J.; Lannutti, B. J.; Wiestner, A.; Woyach, J. A., The Bruton Tyrosine Kinase (BTK) Inhibitor Acalabrutinib Demonstrates Potent On-Target Effects and Efficacy in Two Mouse Models of Chronic Lymphocytic Leukemia. *Clin. Cancer Res.* **2017,** 23, (11), 2831-2841.
3. Li, N.; Sun, Z. J.; Liu, Y.; Guo, M. M.; Zhang, Y. L.; Zhou, D. P.; Zhang, B.; Su, D.; Zhang, S.; Han, J.; Gao, Y. J.; Guo, Y. H.; Wang, Z. W.; Wei, M.; Luo, L. S.; Wang, L., BGB-3111 is a novel and highly selective Bruton's tyrosine kinase (BTK) inhibitor. *Cancer Res.* **2015,** 75.
4. Xu, W.; Song, Y. P.; Li, Z. J.; Yang, S. M.; Liu, L. H.; Hu, Y.; Zhang, W.; Zhou, J. F.; Gao, S. J.; Ding, K. Y.; Zhang, H. L.; Zhu, Z. M.; Wang, S. Q.; Xu, B.; Hu, J. D.; Liu, T.; Ji, C. Y.; Xia, Z. J.; Li, Y.; Wang, X.; Xu, Z. X.; Zhao, R. B.; Zhang, B.; Zhu, A.; Li, J. Y., Safety, Tolerability and Efficacy of Orelabrutinib, Once a Day, to Treat Chinese Patients with Relapsed or Refractory Chronic Lymphocytic Leukemia/Small Cell Leukemia. *Blood* **2019,** 134.
5. Francesco, M. R.; Wong, M.; LaStant, J.; Finkle, D.; Loewenstein, N.; Macsata, R.; Lindstrom, M. M.; Shu, J.; Ton, T.; Zhu, J.; Bradshaw, J. M.; Karr, D.; Hardiman, R.; Goldstein, D. M.; Owens, T. D.; Langrish, C. L., PRN2246, a potent and selective blood brain barrier penetrating BTK inhibitor, exhibits efficacy in central nervous system immunity. *Mult. Scler. J.* **2017,** 23, 511-511.
6. Evans, E. K.; Tester, R.; Aslanian, S.; Karp, R.; Sheets, M.; Labenski, M. T.; Witowski, S. R.; Lounsbury, H.; Chaturvedi, P.; Mazdiyasni, H.; Zhu, Z.; Nacht, M.; Freed, M. I.; Petter, R. C.; Dubrovskiy, A.; Singh, J.; Westlin, W. F., Inhibition of Btk with CC-292 provides early pharmacodynamic assessment of activity in mice and humans. *J. Pharmacol. Exp. Ther.* **2013,** 346, (2), 219-28.
7. Kokabee, L.; Wang, X.; Sevinsky, C. J.; Wang, W. L.; Cheu, L.; Chittur, S. V.; Karimipoor, M.; Tenniswood, M.; Conklin, D. S., Bruton's tyrosine kinase is a potential therapeutic target in prostate cancer. *Cancer Biol. Ther.* **2015,** 16, (11), 1604-15.
8. Kim, Y. Y.; Park, K. T.; Jang, S. Y.; Lee, K. H.; Byun, J. Y.; Suh, K. H.; Lee, Y. M.; Kim, Y. H.; Hwang, K. W., HM71224, a selective Bruton's tyrosine kinase inhibitor, attenuates the development of murine lupus. *Arthritis Res. Ther.* **2017,** 19, (1), 211.
9. Watterson, S.H.; Liu, Q.; Beaudoin, B.M.; Batt, D.G.; Li, L.; Pattoli, M.A.; Skala, S.; Cheng, L.; Obermeier, M.T.; Moore, R.; et al.Discovery of Branebrutinib (BMS-986195): A Strategy for Identifying a Highly Potent and Selective Covalent Inhibitor Providing Rapid in Vivo Inactivation of Bruton's Tyrosine Kinase (BTK). J. Med. Chem. 2019, 62, 3228–3250.

10. Mahajan, S.; Ghosh, S.; Sudbeck, E. A.; Zheng, Y.; Downs, S.; Hupke, M.; Uckun, F. M., Rational design and synthesis of a novel anti-leukemic agent targeting Bruton's tyrosine kinase (BTK), LFM-A13 [alpha-cyano-beta-hydroxy-beta-methyl-N-(2, 5-dibromophenyl)propenamide]. *J. Biol. Chem.* **1999,** 274, (14), 9587-99.
11. Labenski, M.; Chaturvedi, P.; Evans, E.; Mazdiyazni, H.; Sheets, M.; Aslanian, S.; Nacht, M.; Petter, R.; Singh, J.; Westlin, W., In vitro reactivity assessment of covalent drugs targeting Bruton's tyrosine kinase. *Drug Metab. Rev.* **2011,** 43, 140-141.
12. Goess, C.; Harris, C. M.; Murdock, S.; McCarthy, R. W.; Sampson, E.; Twomey, R.; Mathieu, S.; Mario, R.; Perham, M.; Goedken, E. R.; Long, A. J., ABBV-105, a selective and irreversible inhibitor of Bruton's tyrosine kinase, is efficacious in multiple preclinical models of inflammation. *Mod. Rheumatol.* **2019,** 29, (3), 510-522.
13. Caldwell, R. D.; Qiu, H.; Askew, B. C.; Bender, A. T.; Brugger, N.; Camps, M.; Dhanabal, M.; Dutt, V.; Eichhorn, T.; Gardberg, A. S.; Goutopoulos, A.; Grenningloh, R.; Head, J.; Healey, B.; Hodous, B. L.; Huck, B. R.; Johnson, T. L.; Jones, C.; Jones, R. C.; Mochalkin, I.; Morandi, F.; Nguyen, N.; Meyring, M.; Potnick, J. R.; Santos, D. C.; Schmidt, R.; Sherer, B.; Shutes, A.; Urbahns, K.; Follis, A. V.; Wegener, A. A.; Zimmerli, S. C.; Liu-Bujalski, L., Discovery of Evobrutinib: An Oral, Potent, and Highly Selective, Covalent Bruton's Tyrosine Kinase (BTK) Inhibitor for the Treatment of Immunological Diseases. *J. Med. Chem.* **2019,** 62, (17), 7643-7655.
14. Pulz, R.; Angst, D.; Dawson, J.; Gessier, F.; Gutmann, S.; Hersperger, R.; Hinniger, A.; Janser, P.; Koch, G.; Revesz, L.; Vulpetti, A.; Waelchli, R.; Zimmerlin, A.; Cenni, B., Design of Potent and Selective Covalent Inhibitors of Bruton's Tyrosine Kinase Targeting an Inactive Conformation. *ACS Med. Chem. Lett.* **2019,** 10, (10), 1467-1472.
15. Ma, B.; Metrick, C. M.; Gu, C.; Hoemberger, M.; Bajrami, B.; Bame, E.; Huang, J.; Mingueneau, M.; Murugan, P.; Santoro, J. C.; Tang, H.; Wang, T.; Hopkins, B. T., Optimization of a novel piperazinone series as potent selective peripheral covalent BTK inhibitors. *Bioorg. Med. Chem. Lett.* **2022,** 60, 128549.
16. Ma, C.; Li, Q.; Zhao, M.; Fan, G.; Zhao, J.; Zhang, D.; Yang, S.; Zhang, S.; Gao, D.; Mao, L.; Zhu, L.; Li, W.; Xu, G.; Jiang, Y.; Ding, Q., Discovery of 1-Amino-1H-imidazole-5-carboxamide Derivatives as Highly Selective, Covalent Bruton's Tyrosine Kinase (BTK) Inhibitors. *J. Med. Chem.* **2021,** 64, (21), 16242-16270.
17. Sudharshan Eathiraj; Ronald E. Savage; Yi Yu; Brian Schwartz; Sean D. Reiff; Jennifer A. Woyach; Amy J. Johnson; Abbadessa, G., Targeting Ibrutinib-Resistant BTK-C481S Mutation with ARQ 531, a Reversible Non-Covalent Inhibitor of BTK. *Clinical Lymphoma Myeloma & Leukemia*. **2016**, 16.
18. Erickson, R. I.; Schutt, L. K.; Tarrant, J. M.; McDowell, M.; Liu, L.; Johnson, A. R.; Lewin-Koh, S. C.; Hedehus, M.; Ross, J.; Carano, R. A.; Staflin, K.; Zhong, F.; Crawford, J. J.; Zhong, S.; Reif, K.; Katewa, A.; Wong, H.; Young, W. B.; Dambach, D. M.; Misner, D. L., Bruton's Tyrosine Kinase Small Molecule Inhibitors Induce a Distinct Pancreatic Toxicity in Rats. *J. Pharmacol. Exp. Ther.* **2017,** 360, (1), 226-238.

19. Gomez, E. B.; Isabel, L.; Rosendahal, M. S.; Rothenberg, S. M.; Andrews, S. W.; Brandhuber, B. J., Loxo-305, a Highly Selective and Non-Covalent Next Generation BTK Inhibitor, Inhibits Diverse BTK C481 Substitution Mutations. *Blood* **2019,** 134.
20. Hopkins, B. T.; Bame, E.; Bajrami, B.; Black, C.; Bohnert, T.; Boiselle, C.; Burdette, D.; Burns, J. C.; Delva, L.; Donaldson, D.; Grater, R.; Gu, C.; Hoemberger, M.; Johnson, J.; Kapadnis, S.; King, K.; Lulla, M.; Ma, B.; Marx, I.; Magee, T.; Meissner, R.; Metrick, C. M.; Mingueneau, M.; Murugan, P.; Otipoby, K. L.; Polack, E.; Poreci, U.; Prince, R.; Roach, A. M.; Rowbottom, C.; Santoro, J. C.; Schroeder, P.; Tang, H.; Tien, E.; Zhang, F.; Lyssikatos, J., Discovery and Preclinical Characterization of BIIB091, a Reversible, Selective BTK Inhibitor for the Treatment of Multiple Sclerosis. *J. Med. Chem.* **2022,** 65, (2), 1206-1224.
21. Burger, J. A., Bruton's tyrosine kinase (BTK) inhibitors in clinical trials. *Curr. Hematol. Malig. Rep.* **2014,** 9, (1), 44-9.
22. Smith, P. F.; Krishnarajah, J.; Nunn, P. A.; Hill, R. J.; Karr, D.; Tam, D.; Masjedizadeh, M.; Funk, J. O.; Gourlay, S. G., A phase I trial of PRN1008, a novel reversible covalent inhibitor of Bruton's tyrosine kinase, in healthy volunteers. *Br. J. Clin. Pharmacol.* **2017,** 83, (11), 2367-2376.
23. Ma, B.; Bohnert, T.; Otipoby, K. L.; Tien, E.; Arefayene, M.; Bai, J.; Bajrami, B.; Bame, E.; Chan, T. R.; Humora, M.; MacPhee, J. M.; Marcotte, D.; Mehta, D.; Metrick, C. M.; Moniz, G.; Polack, E.; Poreci, U.; Prefontaine, A.; Sheikh, S.; Schroeder, P.; Smirnakis, K.; Zhang, L.; Zheng, F.; Hopkins, B. T., Discovery of BIIB068: A Selective, Potent, Reversible Bruton's Tyrosine Kinase Inhibitor as an Orally Efficacious Agent for Autoimmune Diseases. *J. Med. Chem.* **2020,** 63, (21), 12526-12541.
24. Xu, D.; Kim, Y.; Postelnek, J.; Vu, M. D.; Hu, D. Q.; Liao, C.; Bradshaw, M.; Hsu, J.; Zhang, J.; Pashine, A.; Srinivasan, D.; Woods, J.; Levin, A.; O'Mahony, A.; Owens, T. D.; Lou, Y.; Hill, R. J.; Narula, S.; DeMartino, J.; Fine, J. S., RN486, a selective Bruton's tyrosine kinase inhibitor, abrogates immune hypersensitivity responses and arthritis in rodents. *J. Pharmacol. Exp. Ther.* **2012,** 341, (1), 90-103.
25. Liu, L.; Di Paolo, J.; Barbosa, J.; Rong, H.; Reif, K.; Wong, H., Antiarthritis effect of a novel Bruton's tyrosine kinase (BTK) inhibitor in rat collagen-induced arthritis and mechanism-based pharmacokinetic/pharmacodynamic modeling: relationships between inhibition of BTK phosphorylation and efficacy. *J. Pharmacol. Exp. Ther.* **2011,** 338, (1), 154-63.
26. Pan, Z.; Scheerens, H.; Li, S. J.; Schultz, B. E.; Sprengeler, P. A.; Burrill, L. C.; Mendonca, R. V.; Sweeney, M. D.; Scott, K. C.; Grothaus, P. G.; Jeffery, D. A.; Spoerke, J. M.; Honigberg, L. A.; Young, P. R.; Dalrymple, S. A.; Palmer, J. T., Discovery of selective irreversible inhibitors for Bruton's tyrosine kinase. *ChemMedChem* **2007,** 2, (1), 58-61.
27. Watterson, S. H.; De Lucca, G. V.; Shi, Q.; Langevine, C. M.; Liu, Q.; Batt, D. G.; Beaudoin Bertrand, M.; Gong, H.; Dai, J.; Yip, S.; Li, P.; Sun, D.; Wu, D. R.; Wang, C.; Zhang, Y.; Traeger, S. C.; Pattoli, M. A.; Skala, S.; Cheng, L.; Obermeier, M. T.; Vickery, R.; Discenza, L. N.; D'Arienzo, C. J.; Zhang, Y.; Heimrich, E.; Gillooly, K. M.; Taylor, T. L.; Pulicicchio, C.; McIntyre, K. W.; Galella, M. A.; Tebben, A. J.; Muckelbauer, J. K.; Chang, C.; Rampulla, R.;

Mathur, A.; Salter-Cid, L.; Barrish, J. C.; Carter, P. H.; Fura, A.; Burke, J. R.; Tino, J. A., Discovery of 6-Fluoro-5-(R)-(3-(S)-(8-fluoro-1-methyl-2,4-dioxo-1,2-dihydroquinazolin-3(4H)-yl)-2-methylphenyl)-2-(S)-(2-hydroxypropan-2-yl)-2,3,4,9-tetrahydro-1H-carbazole-8- carboxamide (BMS-986142): A Reversible Inhibitor of Bruton's Tyrosine Kinase (BTK) Conformationally Constrained by Two Locked Atropisomers. *J. Med. Chem.* **2016,** 59, (19), 9173-9200.

28. De Lucca, G. V.; Shi, Q.; Liu, Q.; Batt, D. G.; Beaudoin Bertrand, M.; Rampulla, R.; Mathur, A.; Discenza, L.; D'Arienzo, C.; Dai, J.; Obermeier, M.; Vickery, R.; Zhang, Y.; Yang, Z.; Marathe, P.; Tebben, A. J.; Muckelbauer, J. K.; Chang, C. J.; Zhang, H.; Gillooly, K.; Taylor, T.; Pattoli, M. A.; Skala, S.; Kukral, D. W.; McIntyre, K. W.; Salter-Cid, L.; Fura, A.; Burke, J. R.; Barrish, J. C.; Carter, P. H.; Tino, J. A., Small Molecule Reversible Inhibitors of Bruton's Tyrosine Kinase (BTK): Structure-Activity Relationships Leading to the Identification of 7-(2-Hydroxypropan-2-yl)-4-[2-methyl-3-(4-oxo-3,4-dihydroquinazolin-3-yl)phenyl]- 9H-carbazole-1-carboxamide (BMS-935177). *J. Med. Chem.* **2016,** 59, (17), 7915-35.

B29. uhimschi, A. D.; Armstrong, H. A.; Toure, M.; Jaime-Figueroa, S.; Chen, T. L.; Lehman, A. M.; Woyach, J. A.; Johnson, A. J.; Byrd, J. C.; Crews, C. M., Targeting the C481S Ibrutinib-Resistance Mutation in Bruton's Tyrosine Kinase Using PROTAC-Mediated Degradation. *Biochemistry* **2018,** 57, (26), 3564-3575.

30. Gui, F.; Jiang, J.; He, Z.; Li, L.; Li, Y.; Deng, Z.; Lu, Y.; Wu, X.; Chen, G.; Su, J.; Song, S.; Zhang, Y. M.; Yun, C. H.; Huang, X.; Weisberg, E.; Zhang, J.; Deng, X., A non-covalent inhibitor XMU-MP-3 overrides ibrutinib-resistant Btk(C481S) mutation in B-cell malignancies. *Br. J. Pharmacol.* **2019,** 176, (23), 4491-4509.
