# Supplementary material for: Comparison of Intermolecular Interactions of Irreversible and Reversible Inhibitors with Bruton’s Tyrosine Kinase via Molecular Dynamics Simulations
Source: Molecules. 2022 Nov 2;27(21):7451. doi: 10.3390/molecules27217451 (PMC9655453; doi:10.3390/molecules27217451)
Supplement: Supplementary file 1 [file molecules-27-07451-s001.zip › Supplementary Table S2.pdf]

Table S2: Comparison of inhibitory activity between irreversible (Ibrutinib) and reversible (ARQ531) inhibitors

| Inhibitors | Synonymous | WT/Mut | $IC_{50}$ | References |
|---|---|---|---|---|
| Ibrutinib | PCL-32765 | BTK WT | 0.5 nM | [1] |
| | | | 0.72 nM | [2] |
| | | BTK Cys481 | 9.03 nM | [3] |
| | | | 4.6 nM | [1] |
| ARQ531 | MK-1026 | BTK WT | 0.85 nM | [3] |
| | | BTK Cys481 | 0.39 nM | [3] |


1. Honigberg, L. A.; Smith, A. M.; Sirisawad, M.; Verner, E.; Loury, D.; Chang, B.; Li, S.; Pan, Z.; Thamm, D. H.; Miller, R. A.; Buggy, J. J., The Bruton tyrosine kinase inhibitor PCI-32765 blocks B-cell activation and is efficacious in models of autoimmune disease and B-cell malignancy. *Proc. Natl. Acad. Sci. U. S. A.* **2010,** 107, (29), 13075-80.
2. Johnson, A. R.; Kohli, P. B.; Katewa, A.; Gogol, E.; Belmont, L. D.; Choy, R.; Penuel, E.; Burton, L.; Eigenbrot, C.; Yu, C.; Ortwine, D. F.; Bowman, K.; Franke, Y.; Tam, C.; Estevez, A.; Mortara, K.; Wu, J. S.; Li, H.; Lin, M.; Bergeron, P.; Crawford, J. J.; Young, W. B., Battling Btk Mutants With Noncovalent Inhibitors That Overcome Cys481 and Thr474 Mutations. *ACS Chem. Biol.* **2016,** 11, (10), 2897-2907.
3. Sudharshan Eathiraj; Ronald E. Savage; Yi Yu; Brian Schwartz; Sean D. Reiff; Jennifer A. Woyach; Amy J. Johnson; Abbadessa, G., Targeting Ibrutinib-Resistant BTK-C481S Mutation with ARQ 531, a Reversible Non-Covalent Inhibitor of BTK. *Clinical Lymphoma Myeloma & Leukemia*. **2016**, 16.
